# Causes of Death Among Stillbirths and Children Aged <5 Years in Africa and South Asia — Child Health and Mortality Prevention Surveillance, Seven Countries, 2016–2024

**DOI:** 10.15585/mmwr.ss7506a1

**Published:** 2026-09-10

**Authors:** Zachary J. Madewell, Ziyaad Dangor, Portia C. Mutevedzi, Siobhan L. Johnstone, Sanjay G. Lala, Shabir A. Madhi, Sithembiso Velaphi, Nega Assefa, Meron Kebede, Lola Madrid, Dadi Marami, J. Anthony G. Scott, George Aol, Kitiezo Aggrey Igunza, Hellen Muttai, Peter Nyamthimba Onyango, Peter O. Otieno, Quique Bassat, Marcelino Garrine, Milton Kincardett, Inácio Mandomando, Ariel Nhacolo, Jane Juma, Adama Mamby Keita, Karen L. Kotloff, Samba O. Sow, Milagritos D. Tapia, Afsana Afrin, Shams El Arifeen, Rajib Biswas, Emily S. Gurley, Mohammad Zahid Hossain, Soter Ameh, Ima-Abasi Bassey, Erick Kaluma, Dickens Kowuor, Ikechukwu Udo Ogbuanu, Julu Bhatnagar, Dianna M. Blau, Kevin R. Clarke, Maureen H. Diaz, Michelle Dynes, Jennifer Kasten, Roosecelis B. Martines, Lesley McGee, Diane F. Morof, Elizabeth O’Mara Sage, Pratima L. Raghunathan, Jana Ritter, Wun-Ju Shieh, Allan W. Taylor, Srinivasan Velusamy, Jennifer R. Verani, Ashutosh Wadhwa, Jessica L. Waller, Jonas Winchell, Jacob S. Witherbee, Mary-Claire Worrell, Victor Akelo, Jeffrey P. Koplan, Robert F. Breiman, Cynthia G. Whitney

**Affiliations:** ^1^Global Health Center, CDC, Atlanta, Georgia; ^2^Vaccines and Infectious Diseases Analytics Research Unit, South African Medical Research Council, University of the Witwatersrand, Johannesburg, South Africa; ^3^Emory Global Health Institute, Emory University, Atlanta, Georgia; ^4^Department of Paediatrics and Child Health, Faculty of Health Sciences, University of the Witwatersrand, Johannesburg, South Africa; ^5^Infectious Diseases and Oncology Research Institute, Faculty of Health Sciences, University of the Witwatersrand, Johannesburg, South Africa; ^6^College of Health and Medical Sciences, Haramaya University, Harar, Ethiopia; ^7^Hararghe Health Research, Haramaya University, Harar, Ethiopia; ^8^Department of Infectious Disease Epidemiology and International Health, London School of Hygiene & Tropical Medicine, London, United Kingdom; ^9^Center for Global Health Research, Kenya Medical Research Institute, Kisumu, Kenya; ^10^Liverpool School of Tropical Medicine, Liverpool, United Kingdom; ^11^ISGlobal, Barcelona, Spain; ^12^Centro de Investigação em Saúde de Manhiça, Maputo, Mozambique; ^13^ICREA, Barcelona, Spain; ^14^Instituto Clínic de Medicina y Dermatología, Hospital Clínic de Barcelona, Barcelona, Spain; ^15^Facultat de Medicina i Ciències de la Salut, Universitat de Barcelona, Barcelona, Spain; ^16^Pediatrics Department, Hospital Sant Joan de Déu, Universitat de Barcelona, Barcelona, Spain; ^17^CIBER de Epidemiología y Salud Pública, Instituto de Salud Carlos III, Madrid, Spain; ^18^Global Health and Tropical Medicine, Instituto de Higiene e Medicina Tropical, Universidade NOVA de Lisboa, Lisbon, Portugal; ^19^Instituto Nacional de Saúde, Marracuene, Maputo, Mozambique; ^20^Centre pour le Développement des Vaccins, Ministère de la Santé, Bamako, Mali; ^21^Department of Pediatrics, University of Maryland School of Medicine, Baltimore, Maryland; ^22^ICDDR,B; Dhaka, Bangladesh; ^23^Department of Epidemiology, Johns Hopkins Bloomberg School of Public Health, Baltimore, Maryland; ^24^Child Health and Mortality Prevention Surveillance, The Africa Research Collaborative, Freetown, Sierra Leone; ^25^Department of Community Medicine, Faculty of Clinical Sciences, University of Calabar, Calabar, Nigeria; ^26^Department of Global Health and Population, Harvard T. H. Chan School of Public Health, Harvard University, Boston, Massachusetts; ^27^Department of Pathology, Faculty of Clinical Sciences, University of Calabar, Calabar, Nigeria; ^28^Hubert Department of Global Health, Rollins School of Public Health, Emory University, Atlanta, Georgia; ^29^Infectious Diseases Pathology Branch, National Center for Emerging and Zoonotic Infectious Diseases, CDC, Atlanta, Georgia; ^30^Division of Bacterial Diseases, National Center for Immunization and Respiratory Diseases, CDC, Atlanta, Georgia; ^31^Global Immunization Division, Global Health Center, CDC, Atlanta, Georgia; ^32^Division of Global HIV and TB, Global Health Center, CDC, Atlanta, Georgia; ^33^Division of Foodborne, Waterborne and Environmental Diseases, National Center for Emerging and Zoonotic Infectious Diseases, CDC, Atlanta, Georgia; ^34^Division of State and Local Readiness, Office of Readiness and Response, CDC, Atlanta, Georgia; ^35^CDC Kenya, Kisumu, Kenya; ^36^Child Health and Mortality Prevention Surveillance Network Program Office, Task Force for Global Health, Atlanta, Georgia

## Abstract

**Problem/Condition:**

Sub-Saharan Africa and South Asia have the highest rates of stillbirths and rates of death among children aged <5 years, with many countries in those regions unlikely to meet the 2030 goal of ending preventable deaths among newborns and children aged <5 years. Conventional mortality surveillance (determining cause of death through interviews with family members or caregivers [i.e., verbal autopsy] and vital registration) in areas with high mortality often lacks laboratory confirmation of causes and incompletely identifies maternal contributors and comorbid conditions, providing information that is insufficiently specific to guide prevention efforts.

**Period Covered:**

December 2016–December 2024**.**

**Description of System:**

The Child Health and Mortality Prevention Surveillance (CHAMPS) network generates standardized, laboratory-confirmed data on causes and preventability of deaths among neonates, infants, and children aged <5 years and stillbirths in areas with high mortality. During 2016–2024, CHAMPS operated in seven countries (Bangladesh, Ethiopia, Kenya, Mali, Mozambique, Sierra Leone, and South Africa) through population-defined catchment areas. Eligibility required residence in a CHAMPS catchment area for ≥4 months before death or, for children aged <4 months, since birth. Deaths were investigated using a standardized postmortem approach that included minimally invasive tissue sampling (MITS), laboratory investigations (conventional and molecular microbiology and histopathology), interviews with family members or caregivers (i.e., verbal autopsy), and pediatric and maternal clinical record abstraction. Multidisciplinary determination of cause-of-death (DeCoDe) panels integrated evidence to assign causes (including multiple causes per death) and assess preventability.

**Results:**

During 2016–2024, a total of 18,784 eligible deaths were identified; families consented to CHAMPS enrollment for 15,612 (83.1%) of these deaths. MITS was completed for 9,415 deaths and DeCoDe was completed for 8,500 (90.3% of MITS-investigated deaths); a total of 3,199 (37.6%) were stillbirths; 3,230 (38.0%) were neonatal deaths; and 2,071 (24.4%) were deaths of infants (aged 28 days to <12 months) and children (aged ≥12 to <60 months). Stillbirths were predominantly attributed to perinatal asphyxia or hypoxia (79.1%), often with maternal hypertensive disorders, placental abnormalities, chorioamnionitis, and other medical conditions (e.g., diabetes). Neonatal deaths involved preterm complications (39.7%); asphyxia or hypoxia (37.7%), which often is linked to maternal and intrapartum care; and sepsis (36.5%), commonly resulting from infection with *Klebsiella pneumoniae* and *Acinetobacter baumannii*. Among infants and children, leading causes of death included lower respiratory infections (37.4%), sepsis (36.9%), malnutrition (27.3%), malaria (22.1%), and diarrheal disease (17.3%). Multiple conditions in the causal chain were common among neonates (44.3% with two or more conditions) and infants and children (67.9% with two or more conditions). Postmortem anthropometry indicated high levels of moderate or severe undernutrition among infants and children; among those with available measurements, nonmutually exclusive anthropometric indicators included underweight (61.3%), wasting (61.3%), and stunting (43.0%), according to the World Health Organization’s Child Growth Standards. Among neonatal, infant, and child deaths (excluding stillbirths), infection contributed to 3,146 (59.3%) deaths. Among neonatal, infant, and child deaths with one or more pathogens identified in the causal chain (n = 2,749), 44.5% were polymicrobial, with gram-negative bacteria predominating. Main maternal conditions, most commonly placental complications and hypertensive disorders of pregnancy, were assigned for 71.1% of stillbirths and 58.2% of neonatal deaths. Of 7,558 deaths with a preventability assessment, 6,103 (80.7%) were considered preventable or possibly preventable through improvements in already available maternal, newborn, and child health interventions; primary opportunities included strengthened antenatal care, obstetric management, and infection control.

**Interpretation:**

CHAMPS complements conventional mortality surveillance by providing standardized, laboratory-confirmed, postmortem evidence on causes and preventability of stillbirths and of deaths among neonates, infants, and children aged <5 years across areas with high mortality. Findings demonstrate that preventable infections, suboptimal antenatal care, intrapartum complications, preterm birth, and malnutrition account for most deaths and also involve maternal and health care system factors. The predominance of infections caused by gram-negative bacteria highlights the need to strengthen infection prevention and control and to develop preventive tools, including vaccines.

**Public Health Action:**

Four of every five deaths were potentially preventable with timely implementation of established maternal, newborn, and child health interventions. CHAMPS demonstrates that high-precision postmortem MITS is feasible in resource-constrained settings and provides data critical to improve maternal and child health planning, practice, and policy. Certain components of CHAMPS (e.g., standardized MITS training, targeted diagnostics, and multidisciplinary review) could be adapted to strengthen routine surveillance and mortality review systems where feasible.

## Introduction

In 2023, an estimated 4.9 million deaths among children aged <5 years and 1.9 million stillbirths occurred worldwide; approximately 80% were in sub-Saharan Africa and South Asia, where deaths are likely underestimated and accurate cause-of-death determination remains challenging (*1*). Despite progress in reducing deaths, limited access to high-quality obstetric and pediatric care, health care workforce shortages, and inadequate diagnostic capacity contribute to high rates of preventable fetal, neonatal, infant, and child deaths in these regions (*2*,*3*). Accurate identification of underlying causes and contributing conditions is essential for understanding causal pathways and guiding prevention strategies. In many low- and middle-income countries (LMICs), death registration and medical certification remain incomplete, and many deaths occur outside health care facilities or shortly after arrival, with little opportunity for diagnostic evaluation (*4*). Conventional methods for cause-of-death attribution in LMICs, including verbal autopsy, rely largely on interviews with family members or other lay respondents and are designed primarily to assign a single probable underlying cause on the basis of limited information (*5*,*6*). These approaches generally do not identify multiple coexisting or contributory conditions. As a result, routine mortality data often record only one underlying cause and, without diagnostic confirmation, might be nonspecific or imprecise and obscure contributory factors relevant to prevention.

The Child Health and Mortality Prevention Surveillance (CHAMPS) network was established in 2016 to generate accurate cause-of-death data and guide prevention strategies in high-mortality settings. CHAMPS investigates deaths among neonates, infants, and children aged <5 years and stillbirths using standardized postmortem methods, including laboratory evaluation and minimally invasive tissue sampling (MITS) (*7*–*9*). After a death is identified, CHAMPS teams collect tissue and body fluid specimens for microbiologic, molecular, and histopathologic analyses, enabling identification of infectious, nutritional, perinatal, and other causes of death while maintaining cultural acceptability (*10*–*12*). These findings are integrated with verbal autopsy, clinical records, and contextual information on social determinants and health care system factors. At each site, multidisciplinary determination of cause of death (DeCoDe) panels review all available data using standardized World Health Organization (WHO) *International Classification of Diseases* (*ICD*) frameworks to assign underlying, antecedent, and immediate causes and to identify contributing maternal conditions, comorbidities, and health care system factors across the pregnancy-to-childhood continuum (*13*,*14*).

During 2016–2024, CHAMPS operated in defined catchment areas in Bangladesh and six African countries: Ethiopia, Kenya, Mali, Mozambique, Sierra Leone, and South Africa. CHAMPS was implemented through partnerships with national research and public health institutions and coordinated through the CHAMPS Program Office in collaboration with CDC and the Task Force for Global Health’s Public Health Informatics Institute. CDC provided scientific leadership, core mortality surveillance methods, laboratory approaches, and ongoing pathology and diagnostic support (*15*,*16*). CHAMPS findings were routinely shared with ministries of health and other stakeholders to support prevention activities and contribute to initiatives related to antimicrobial resistance, vaccine-preventable diseases, and maternal and child health surveillance (*13*,*17*,*18*).

This report summarizes surveillance findings during December 2016–December 2024 across CHAMPS catchment areas in seven countries and describes CHAMPS methods, laboratory systems, and findings on causes and preventability of stillbirths and deaths among neonates, infants, and children aged <5 years. Building on approximately 100 CHAMPS publications, including reports focused on specific countries, age groups, and conditions (*19*–*22*), this report highlights cross-site patterns, shared causal pathways, and health care system gaps that can guide improvements in mortality surveillance and strategies to reduce preventable deaths.

## Methods

### Surveillance System Overview

**FIGURE 1:**
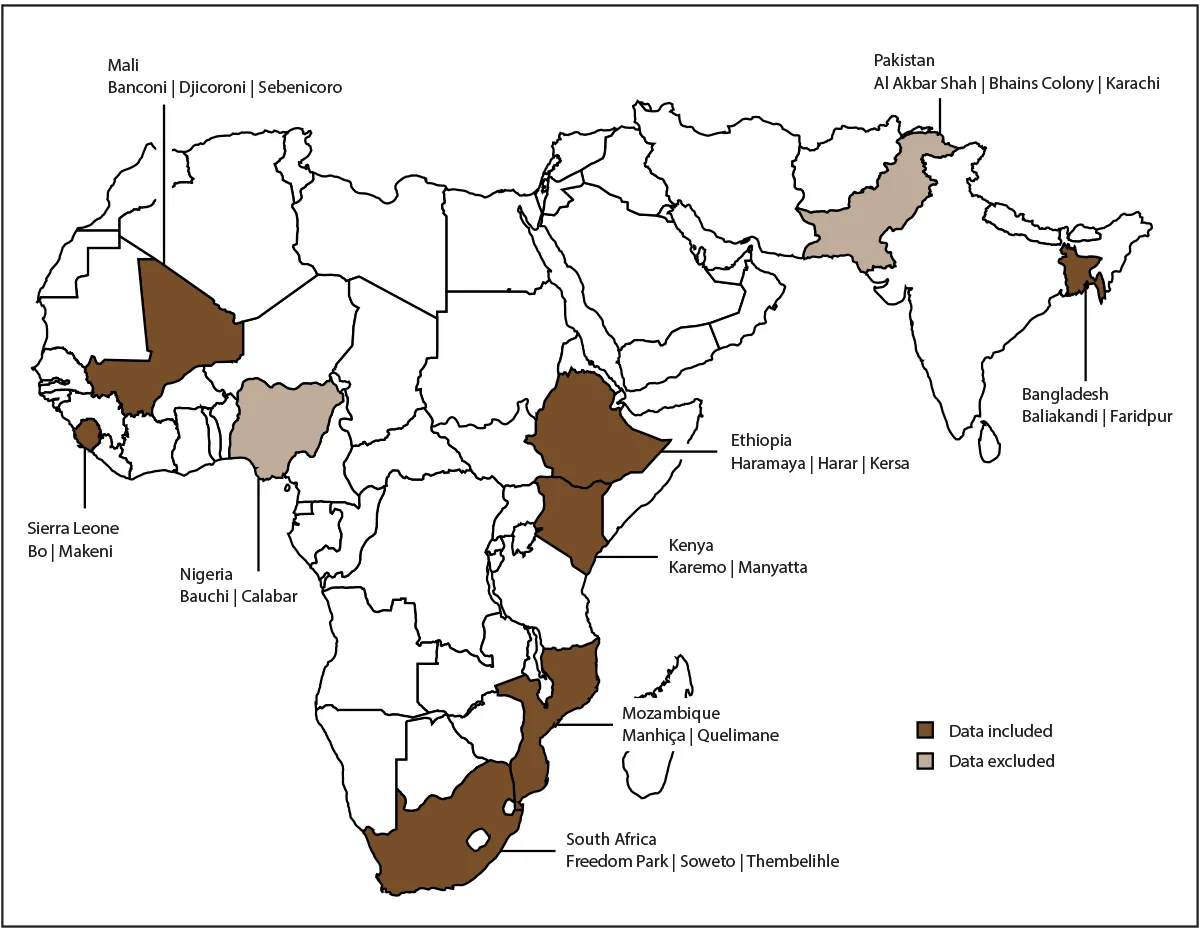
Child Health and Mortality Prevention Surveillance network sites, 2016–2024* * Surveillance at each site began at different times across countries and sites. Nigeria (Bauchi and Calabar) and Pakistan (Al Akbar Shah, Bhains Colony, and Karachi) were added to the Child Health and Mortality Prevention Surveillance network in 2023 and were in the start-up phase (i.e., limited case accrual and determination of cause-of-death reviews pending) during this analytic period; thus, no data from these sites are included.

CHAMPS sites were selected through a multistage process on the basis of mortality rates, geographic representation, existing research infrastructure, and government commitment (*7*). During 2016–2024, surveillance sites operated in Bangladesh (Baliakandi and Faridpur), Ethiopia (Haramaya, Harar, and Kersa), Kenya (Karemo and Manyatta), Mali (Banconi, Djicoroni, and Sebenicoro), Mozambique (Manhiça and Quelimane), Sierra Leone (Bo and Makeni), and South Africa (Freedom Park, Soweto, and Thembelihle) (Figure 1). Site leadership teams were based at national research and public health institutions, including ICDDR,B (Dhaka, Bangladesh); College of Health and Medical Sciences, Haramaya University (Harar, Ethiopia); Kenya Medical Research Institute-Center for Global Health Research (Kisumu, Kenya); Centre pour le Développement des Vaccins-Mali (Bamako, Mali); Centro de Investigação em Saúde de Manhiça (Maputo, Mozambique); The Africa Research Collaborative for Health (Freetown, Sierra Leone); and Vaccines and Infectious Diseases Analytics Research Unit of the South African Medical Research Council, University of the Witwatersrand (Johannesburg, South Africa). Pakistan (Al Akbar Shah, Bhains Colony, and Karachi) and Nigeria (Bauchi and Calabar) joined during 2023–2024, but their data are excluded because those sites were in the early start-up phase of implementation.

Surveillance was implemented in collaboration with ministries of health within population-defined catchment areas with approximately 70,000–2,000,000 residents. Deaths were identified through health care facilities, community-based death-reporting networks, demographic surveillance systems, mortuaries, and civil registration offices (*5*,*23*). Most CHAMPS sites have public health and demographic surveillance systems that provide vital denominators and mortality notification frameworks (*23*). Ongoing community engagement supported culturally appropriate implementation, disclosure of cause-of-death findings to families, and dissemination of results to community leaders and public health officials. Sites also routinely shared data with health authorities to guide public health action (*5*,*23*,*24*). Network coordination, protocol standardization, and data management were conducted through the CHAMPS Program Office at Emory University (2016–2025) in partnership with CDC and the Task Force for Global Health’s Public Health Informatics Institute; in July 2025, the CHAMPS Program Office transitioned to the Task Force for Global Health. Surveillance protocols, laboratory procedures, data systems, and analytic workflows were standardized across sites (*14*). Detailed methods for CHAMPS field procedures, laboratory platforms (including custom syndromic TaqMan array cards), pathology protocols, and quality assurance have been published elsewhere (*5*,*11*–*16*,*23*,*24*). Additional information is available (CHAMPS resources).

### Community Engagement and Feasibility

Before implementation, social and behavioral sciences (SBS) teams conducted formative research to understand local cultural, religious, and social norms related to death and burial and to develop community engagement and consent procedures (*5*,*12*,*24*). Activities included participatory meetings with community, traditional, and religious leaders; information sessions for caregivers; and monitoring of community concerns, misinformation, and rumors about MITS so they could be identified and addressed promptly. During surveillance, SBS staff members supported bereavement-sensitive consent, coordinated with local leaders to respect funeral timing and practices, and maintained community feedback mechanisms to identify barriers and adapt messages and procedures. These activities began before introduction of MITS and continued throughout implementation to support acceptability and guide operational adjustments. After DeCoDe review, families were offered an in-person results disclosure visit during which a clinician, often accompanied by SBS staff members, explained findings in plain language, provided grief counseling and psychosocial support, answered questions, and facilitated referral for clinical or mental health services when indicated. At many sites, families were offered more than one follow-up visit, and disclosure visits also provided opportunities to address identified preventable factors and connect families with relevant health care services.

### Case Eligibility and Enrollment

CHAMPS enrolled stillbirths whose mothers met the catchment area–residency requirement and deaths among neonates, infants, and children aged <5 years who had lived in the catchment area for at least 4 months before death or since birth if aged <4 months (*5*). Eligibility for MITS required timely notification so that postmortem specimen collection could occur within 24 hours of death, or within 72 hours if the body had been refrigerated (*5*). Stillbirths were defined as fetal deaths at ≥28 weeks’ gestation or a birth weight of ≥1,000 g and no signs of life at delivery (*25*). Neonatal deaths were defined as deaths among live-born infants aged <28 days (*9*), and infant and child deaths were defined as deaths among children aged 28 days to <60 months (*8*).

Deaths were identified through notifications from health care facilities, including maternity wards, pediatric and emergency units, and mortuaries, and through mobile-platform reports submitted by designated community death reporters (*12*). Deaths were classified as facility deaths if they occurred in health care facilities, including referral hospitals, community hospitals, and clinics; deaths occurring outside health care facilities were classified as community deaths. Site teams contacted families to obtain consent for MITS and related data collection, including clinical record abstraction and verbal autopsy. When MITS was declined or was not feasible, such as after delayed notification or because the body had not been refrigerated, families were invited to participate in a non-MITS pathway that included verbal autopsy and, when available, clinical record abstraction. Community-based mobile teams were established to support timely local response to deaths that occurred in homes. Enrollment stopped at all sites during the early months of the COVID-19 pandemic and restarted at different times as communities lifted restrictions.

### Minimally Invasive Tissue Sampling

Before specimen collection, postmortem anthropometric measurements, including weight, length, and mid-upper arm circumference (MUAC), were obtained using calibrated scales, length boards, and MUAC tapes. z scores for weight-for-age (WAZ), length-for-age (LAZ), weight-for-length (WLZ), and MUAC-for-age (MUACZ) were calculated according to WHO Child Growth Standards to support standardized cross-site comparisons (*26*,*27*). z scores were categorized as normal (≥ −2 SD), moderate (−3 SD to < −2 SD), and severe (< −3 SD). Indicators of undernutrition were defined as underweight (WAZ < −2 SD), stunting (LAZ < −2 SD), and wasting (WLZ < −2 SD or MUACZ < −2 SD).

MITS protocols were age-specific and standardized across sites (*11*). For all deaths, specimens included whole blood, cerebrospinal fluid (CSF), nasopharyngeal and rectal swabs, and tissue biopsies from the lung, liver, and brain. For stillbirths and neonatal deaths, placental disc, membranes, and umbilical cord specimens also were collected to assess maternal-fetal and perinatal conditions (*5*). Specimen collection was performed by trained personnel, including pathologists, clinicians, and dedicated nonphysician MITS technicians, using sterile technique and 14- to 16-gauge biopsy needles (*11*,*16*).

Each site maintained a biosafety level 2 mortuary laboratory for specimen processing (*16*). Whole blood and CSF were collected in ethylenediaminetetraacetic acid (EDTA) tubes; specimens for culture and molecular testing were stored at 2°C–8°C (32.6°F–46.4°F) and transported within 4 hours to the appropriate laboratory for analysis. Formalin-fixed specimens were used for histopathologic evaluation. Standardized procedures were supported through international training, quality-assurance review, and telepathology mentorship from CDC pathology experts (*16*).

### Laboratory Diagnostics

CHAMPS laboratories conducted standardized postmortem diagnostic testing to identify infectious and noninfectious causes of death (*15*,*28*,*29*). Blood and CSF specimens underwent bacterial culture testing; in South Africa, lung tissue also was cultured for bacteria and fungi, and blood and CSF were cultured for fungi. Molecular testing was performed on lung tissue, blood, CSF, nasopharyngeal and oropharyngeal swabs, and rectal swabs using CDC-developed multiplex syndromic TaqMan array cards that detected approximately 100 bacterial, viral, fungal, and parasitic targets (*15*). Because certain TaqMan array card targets are shared by *Escherichia coli* and *Shigella* spp./enteroinvasive *E. coli*, these detections are reported as “*E. coli/Shigella* spp.”; detailed methods for pathotype differentiation have been described (*30*). *Mycobacterium tuberculosis* was detected by GeneXpert MTB/RIF Ultra, and malaria infection was confirmed by microscopy, rapid diagnostic testing, or molecular assays (*13*,*15*,*31*). HIV infection was assessed by qualitative polymerase chain reaction (PCR) for viral RNA or DNA using whole blood or dried blood spot specimens (*32*).

Histopathologic evaluation was performed at site laboratories and CDC reference laboratories (*16*). Hematoxylin and eosin staining was used to characterize tissue injury and inflammation. When histopathologic findings or clinical context indicated the need for additional evaluation, ancillary testing was performed on a case-by-case basis and guided by CHAMPS syndromic algorithms and molecular and microbiologic results. Ancillary testing included special stains, immunohistochemistry, formalin-fixed paraffin-embedded tissue–based 16S ribosomal RNA PCR with sequencing, targeted PCR or reverse–transcription PCR, and other site-specific assays. Telepathology enabled remote case review using whole-slide imaging and the CHAMPS telepathology platform (slides scanned at sites using NanoZoomer-SQ digital slide scanners and at CDC using a Leica Aperio AT2, then uploaded to the CHAMPS central server for shared viewing and annotation). CDC-coordinated quality assurance, including standardized histopathology checklists, diagnostic algorithms, and telepathology-based case review, supported consistency in pathology interpretation across site and reference laboratories (*16*).

### Verbal Autopsy

When consent was provided by a parent, caregiver, or other close family member, trained interviewers administered a locally translated WHO 2016 verbal autopsy questionnaire for deaths occurring in health care facilities or the community (*5*,*33*). The questionnaire was adapted for CHAMPS to include site identifiers and updated content, skip logic, and corrected units of measurement. Interviews were conducted after burial and at the family’s convenience, typically 2–4 weeks after death and usually within 12 weeks, to balance mourning practices with recall accuracy.

### Clinical and Maternal Record Abstraction

For deaths occurring in health care facilities, clinical and maternal records were abstracted using standardized abstraction forms to collect demographic characteristics, admission diagnoses, vital signs, anthropometric measurements, laboratory findings, treatments, and clinical course (*5*,*13*). Neonatal abstraction forms included labor and delivery circumstances, Apgar scores, resuscitation, early feeding, and neonatal complications. Stillbirth abstraction forms included maternal age, parity, antenatal care visits, ultrasound findings, hypertensive disorders, maternal infections, and intrapartum events.

### Determination of Cause of Death

Multidisciplinary DeCoDe panels, including clinicians, pediatricians, obstetricians, pathologists, microbiologists, and epidemiologists, reviewed laboratory, pathology, clinical, maternal, and verbal autopsy data compiled in standardized electronic dossiers. Panels assigned causes of death using WHO *ICD,* *Tenth Revision* (*ICD-10*) and *ICD for Perinatal Mortality* (*ICD-PM*) codes, which classify perinatal deaths by timing and links them to five maternal condition groups: M1, complications of the placenta, cord, or membranes; M2, maternal complications of pregnancy; M3, other complications of labor and delivery; M4, maternal medical or surgical conditions; and M5, no maternal condition identified. A phased transition to *ICD,* *Eleventh Revision* began in 2022. For this report, maternal conditions were summarized by using standard CHAMPS groupings that map DeCoDe-assigned *ICD* diagnoses to the *ICD-PM* maternal condition framework and predefined subcategories, consistent with previous CHAMPS perinatal analyses. Each DeCoDe record could include approximately 120 laboratory results and thousands of discrete clinical, maternal, pathology, and verbal autopsy data elements per case (*14*).

Panelists evaluated the sequence of events leading to death, identified one underlying cause and, when applicable, identified one or more antecedent causes and an immediate cause (*8*,*13*). The underlying cause was the condition that initiated the chain of events leading to death; antecedent causes were intermediate conditions in that chain, and the immediate cause was the final condition directly resulting in death. Panels integrated laboratory and pathology findings with clinical and contextual information to determine whether identified pathogens or other conditions were causal and to identify deaths in which multiple conditions contributed (*17*,*30*). When deciding whether to include a condition in the causal chain, panelists applied counterfactual reasoning: they considered whether the death would have occurred, when it occurred, in the absence of that condition (*13*). Network quality assurance included periodic cross-site review of a small subset of cases from each site; results were analyzed centrally to assess interpanel concordance and guide retraining and revisions to diagnosis standards (*14*). Any diagnosis classified as an underlying, antecedent, or immediate cause was counted as a cause; therefore, cause-specific proportions reflect multicausal attribution and can sum to >100%.

### Preventability Assessment

For each case with a determined cause of death, DeCoDe panels completed a structured preventability assessment (*3*,*14*). Panelists classified a death as 1) preventable when they assessed that one or more plausible changes in health care system performance, access to care, or family response could have averted the death; 2) possibly preventable under certain circumstances when they assessed that the death might have been averted only under specified circumstances; or 3) not preventable when no such change was considered likely to avert the death. Classifications were assigned within the context of services, resources, and policies available in the facility and community and focused on circumstances immediately surrounding the death rather than broader political, financial, or social determinants. As part of the preventability assessment, panelists could identify one or more standardized health care system improvement categories and subcategories, including antenatal care, obstetric care, pediatric clinical management and quality of care, health care–seeking behavior, health education, infection prevention and control, nutritional support, HIV infection prevention and treatment, vaccination, and transportation. These categories were derived from early DeCoDe free-text recommendations and subsequently applied across sites. A subset of DeCoDe-reviewed cases underwent secondary cross-site review as a quality-control measure; however, the interrater reliability specifically for preventability classifications was not calculated. Individual preventability recommendations were shared with participating families when relevant for the health of the parents (e.g., pregnancy-related conditions that might reoccur) or other children (e.g., malnutrition or vaccine-preventable conditions); aggregate preventability findings and recommendations were shared with health care facilities, communities, ministries of health, and other public health stakeholders to guide locally appropriate prevention activities.

### Data Management and Quality Assurance

Data were collected by using standardized electronic systems, including Research Electronic Data Capture (REDCap [version 16.1.4; Vanderbilt University]) and LabKey, with embedded range and logic checks, automated audit trails, and secure data transfer protocols (*11*,*14*). The CHAMPS Program Office conducted routine audits and cross-site data harmonization. Deidentified data were stored in a centralized repository under standardized quality-control procedures. CHAMPS maintained a centralized, web-accessible global data repository governed by a data governance committee; access was role based, and deidentified datasets could be made available after registration and approval, with additional protections for datasets that included potentially identifying variables.

### Ethics

Activities were approved by institutional review boards or national ethics committees in each participating country and by Emory University. This activity was reviewed by CDC and conducted consistent with applicable federal law and CDC policy.* CHAMPS used tiered informed consent to balance scientific value with family autonomy and local practices. Trained staff members proficient in the local language approached families soon after death notification to explain procedures and obtain written informed consent before enrollment and data collection. Community engagement and formative social and behavioral research guided the way that teams explained MITS, approached families during bereavement, coordinated with funeral and burial practices, and adapted site-specific consent procedures while preserving voluntary participation. Ongoing feedback from families, community leaders, and local teams was used to identify concerns and refine messaging, timing of family approach, and data-collection procedures. Materials supporting cause-of-death determination (e.g., clinical documents and pathology images) were handled under approved protocols (*14*). Activities were coordinated with community leaders to respect burial practices and support timely release of the body. Data were deidentified, stored securely, and shared under data-use agreements with ministries of health.

## Results

### Enrollment and MITS Completion

**FIGURE 2:**
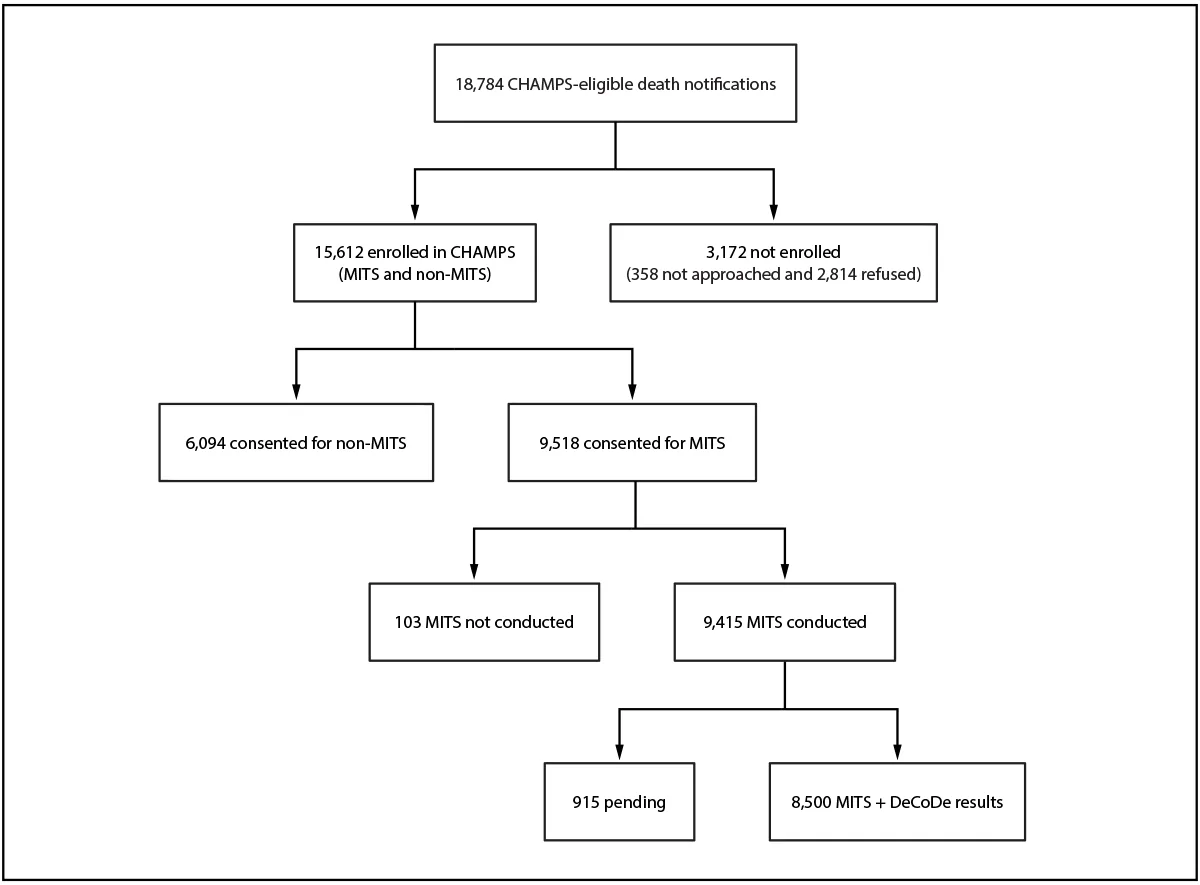
Death notifications, enrollment, minimally invasive tissue sampling,* and determination of cause-of-death review among stillbirths, neonates, infants, and children aged <5 years in Africa and South Asia — Child Health and Mortality Prevention Surveillance network, seven countries,^†^ 2016–2024^§^ **Abbreviations:** CHAMPS = Child Health and Mortality Prevention Surveillance; DeCoDe = determination of cause of death; MITS = minimally invasive tissue sampling. * Non-MITS refers to verbal autopsy and, when available, clinical record abstraction without MITS. ^†^ Bangladesh, Ethiopia, Kenya, Mali, Mozambique, Sierra Leone, and South Africa. ^§^ N = 8,500 deaths with completed MITS and DeCoDe results.

During December 2016–December 2024, a total of 18,784 eligible stillbirths and deaths among neonates, infants, and children aged <5 years were reported across seven CHAMPS countries (Bangladesh, Ethiopia, Kenya, Mali, Mozambique, Sierra Leone, and South Africa); 15,612 (83.1%) of these deaths were enrolled (Figure 2) (Supplementary Table 1). Consent for MITS was obtained for 9,518 deaths, and MITS analysis was completed for 9,415 (98.9%) of these deaths. A total of 8,500 (90.3%) deaths with completed MITS analyses also underwent complete DeCoDe review; these 8,500 deaths were included in the analysis.

### Demographic, Clinical, and Anthropometric Characteristics

Among the 8,500 deaths included in the analysis, 3,199 (37.6%) were stillbirths, 3,230 (38.0%) were neonatal deaths, and 2,071 (24.4%) were infant or child deaths (Table 1). The proportion of stillbirths ranged from 57.7% (761 of 1,318) in Ethiopia to 25.6% (303 of 1,183) in Sierra Leone. Males accounted for 55.5% of deaths. Overall, 88.2% of deaths occurred in health care facilities and 11.8% in the community. Facility deaths accounted for most deaths in all sites, ranging from 74.3% in Kenya and 83.7% in Ethiopia to >90% in Bangladesh, Mozambique, and Sierra Leone. Median duration of stay in a health care facility before death (excluding stillbirths) was 31 hours (IQR = 9–94); country-specific medians ranged from 18 hours in Kenya to 81 hours in South Africa. The overall median interval from death to MITS completion was 9 hours (IQR = 3–17); by country, the median interval ranged from 1 hour in Bangladesh to 23 hours in South Africa.

**TABLE 1:** Number and percentage* of stillbirths and deaths among infants, neonates, and children aged <5 years in Africa and South Asia, by country, demographic characteristics, and clinical characteristics — Child Health and Mortality Prevention Surveillance network, seven countries, 2016–2024

Characteristic	Bangladesh	Ethiopia	Kenya	Mali	Mozambique	Sierra Leone	South Africa	Total
	No. (%)	No. (%)	No. (%)	No. (%)	No. (%)	No. (%)	No. (%)	No. (%)
**Age group (n = 8,500)**								
Stillbirth	543 (52.1)	761 (57.7)	381 (28.9)	276 (36.8)	561 (37.0)	303 (25.6)	374 (27.2)	**3,199 (37.6)**
Neonate (≤27 days)	485 (46.5)	415 (31.5)	380 (28.8)	305 (40.7)	655 (43.2)	374 (31.6)	616 (44.9)	**3,230 (38.0)**
Infant (28 days to <12 months)	12 (1.2)	63 (4.8)	294 (22.3)	102 (13.6)	127 (8.4)	216 (18.3)	264 (19.2)	**1,078 (12.7)**
Child (12 to <60 months)	3 (0.3)	79 (6.0)	264 (20.0)	66 (8.8)	172 (11.4)	290 (24.5)	119 (8.7)	**993 (11.7)**
**Sex (n = 8,498)**								
Female	465 (44.6)	619 (47.0)	601 (45.6)	343 (45.9)	660 (43.6)	518 (43.8)	568 (41.4)	**3,774 (44.4)**
Male	577 (55.3)	698 (53.0)	717 (54.4)	404 (54.0)	852 (56.3)	665 (56.2)	801 (58.3)	**4,714 (55.5)**
Indeterminate or ambiguous	1 (0.1)	1 (0.1)	1 (0.1)	1 (0.1)	2 (0.1)	0 (—)	4 (0.3)	**10 (0.1)**
**Location of death (n = 8,499)**								
Community	37 (3.5)	215 (16.3)	339 (25.7)	92 (12.3)	93 (6.1)	69 (5.8)	161 (11.7)	**1,006 (11.8)**
Facility	1,006 (96.5)	1,103 (83.7)	980 (74.3)	657 (87.7)	1,422 (93.9)	1,114 (94.2)	1,211 (88.3)	**7,493 (88.2)**
**Median duration of hospital stay, hrs (IQR) (n = 3,760)^†^**	22 (7–51)	25 (5–66)	18 (7–50)	33 (10–83)	25 (8–64)	34 (10–107)	81 (24–217)	**31 (9–94)**
**Median time from death to MITS, hrs (IQR) (n = 8,500)**	1 (1–2)	3 (1–7)	16 (9–22)	8 (3–14)	12 (5–18)	7 (3–13)	23 (14–34)	**9 (3–17)**
**Number of causal conditions identified in each death (n = 8,500)**								
1	743 (71.2)	764 (58.0)	984 (74.6)	443 (59.1)	1,151 (76.0)	701 (59.3)	665 (48.4)	**5,451 (64.1)**
2	221 (21.2)	252 (19.1)	218 (16.5)	170 (22.7)	242 (16.0)	247 (20.9)	288 (21.0)	**1,638 (19.3)**
3	67 (6.4)	158 (12.0)	92 (7.0)	84 (11.2)	95 (6.3)	159 (13.4)	210 (15.3)	**865 (10.2)**
≥4	12 (1.2)	144 (10.9)	25 (1.9)	52 (6.9)	27 (1.8)	76 (6.4)	210 (15.3)	**546 (6.4)**
Median (IQR)	1 (1–2)	1 (1–2)	1 (1–2)	1 (1–2)	1 (1–1)	1 (1–2)	2 (1–3)	**1 (1–2)**
**Weight-for-age z score (n = 2,026)^§^**								
Normal (≥ −2 SD)	0 (—)	16 (11.3)	228 (41.0)	51 (33.6)	130 (43.9)	212 (42.0)	148 (41.0)	**785 (38.7)**
Moderate underweight (−3 SD to <−2 SD)	2 (14.3)	16 (11.3)	83 (14.9)	31 (20.4)	39 (13.2)	109 (21.6)	41 (11.4)	**321 (15.8)**
Severe underweight (< −3 SD)	12 (85.7)	110 (77.5)	245 (44.1)	70 (46.1)	127 (42.9)	184 (36.4)	172 (47.6)	**920 (45.4)**
Median (IQR)	−5.4 (−7.0 to −3.4)	−4.8 (−5.8 to −3.3)	−2.5 (−4.4 to −1.1)	−2.8 (−4.9 to −1.6)	−2.4 (−4.2 to −1.0)	−2.3 (−3.7 to −1.1)	−2.6 (−5.0 to −0.6)	**−2.6 (−4.5 to −1.2)**
**Length-for-age z score (n = 2,008)^§^**								
Normal (≥ −2 SD)	7 (46.7)	18 (13.0)	358 (64.4)	93 (61.2)	124 (43.1)	337 (67.8)	207 (57.2)	**1,144 (57.0)**
Moderate stunting (−3 SD to <−2 SD)	2 (13.3)	15 (10.9)	86 (15.5)	19 (12.5)	52 (18.1)	79 (15.9)	41 (11.3)	**294 (14.6)**
Severe stunting (< −3 SD)	6 (40.0)	105 (76.1)	112 (20.1)	40 (26.3)	112 (38.9)	81 (16.3)	114 (31.5)	**570 (28.4)**
Median (IQR)	−2.4 (−5.6 to −0.6)	−4.7 (−6.2 to −3.1)	−1.3 (−2.7 to 0)	−1.3 (−3.1 to 0)	−2.5 (−4.2 to −0.8)	−1.2 (−2.4 to −0.1)	−1.5 (−4.2 to 0.6)	**−1.6 (−3.4 to −0.2)**
**Weight-for-length z score (n = 1,918)^§^**								
Normal (≥ −2 SD)	0 (—)	53 (40.5)	211 (39.3)	49 (31.6)	162 (57.7)	220 (44.4)	163 (52.6)	**858 (44.7)**
Moderate wasting (−3 SD to <−2 SD)	1 (11.1)	24 (18.3)	105 (19.6)	27 (17.4)	34 (12.1)	94 (19.0)	40 (12.9)	**325 (16.9)**
Severe wasting (< −3 SD)	8 (88.9)	54 (41.2)	221 (41.2)	79 (51.0)	85 (30.2)	181 (36.6)	107 (34.5)	**735 (38.3)**
Median (IQR)	−4.7 (−5.4 to −3.8)	−2.5 (−4.1 to −1.1)	−2.5 (−4.2 to −1.2)	−3.0 (−4.1 to −1.8)	−1.5 (−3.3 to 0.1)	−2.3 (−3.7 to −1.0)	−1.8 (−3.9 to 0)	**−2.3 (−3.9 to −0.8)**
**Mid-upper arm circumference-for-age z score (n = 1,689)^§^**								
Normal (≥ −2 SD)	1 (16.7)	18 (15.1)	259 (52.4)	51 (46.4)	140 (55.3)	285 (63.5)	177 (68.6)	**931 (55.1)**
Moderate malnutrition (−3 SD to <−2 SD)	2 (33.3)	18 (15.1)	69 (14.0)	21 (19.1)	31 (12.3)	68 (15.1)	21 (8.1)	**230 (13.6)**
Severe malnutrition (< −3 SD)	3 (50.0)	83 (69.7)	166 (33.6)	38 (34.5)	82 (32.4)	96 (21.4)	60 (23.3)	**528 (31.3)**
Median (IQR)	−3.3 (−6.4 to −2.2)	−4.2 (−5.7 to −2.7)	−1.9 (−3.6 to −0.6)	−2.1 (−3.9 to −0.7)	−1.7 (−3.8 to −0.5)	−1.4 (−2.7 to −0.3)	−0.9 (−2.9 to 0.6)	**−1.7 (−3.6 to −0.4)**
**Severe wasting^¶^ (n = 1,987)^§^**								
Yes	10 (83.3)	91 (66.9)	251 (45.6)	80 (51.6)	125 (43.0)	189 (37.7)	138 (40.4)	**884 (44.5)**
No	2 (16.7)	45 (33.1)	299 (54.4)	75 (48.4)	166 (57.0)	312 (62.3)	204 (59.6)	**1,103 (55.5)**
**Total**	**1,043 (100)**	**1,318 (100)**	**1,319 (100)**	749 (100)**	**1,515 (100)**	**1,183 (100)**	**1,373 (100)**	**8,500 (100)**

**Abbreviation:** MITS = minimally invasive tissue sampling.

* Percentages might not sum to 100% because of rounding.

^†^ Excludes stillbirths.

^§^ Excludes stillbirths and neonates.

^¶^ Weight-for-length z score or mid-upper arm circumference-for-age z score < −3 SD

** Mali suspended MITS in January 2024; counts therefore reflect reduced enrollment during 2024.

Postmortem anthropometry of bodies indicated a high prevalence of undernutrition among infants and children, with country-level variation; these measures did not necessarily correspond to malnutrition being included in the causal chain. Among infants and children with available measurements, 61.3% (1,241 of 2,026) were moderately or severely underweight, 43.0% (864 of 2,008) were stunted, and 61.3% (1,218 of 1,987) were wasted (Table 1). Severe underweight was most frequent in Ethiopia (77.5%), severe stunting in Ethiopia (76.1%) and Mozambique (38.9%), and severe wasting in Ethiopia (66.9%), Mali (51.6%), and Kenya (45.6%).

### Causes of Death

Multiple conditions were commonly identified in the causal chain leading to death, particularly among neonates (44.3% with two or more conditions) and infants and children (67.9% with two or more conditions). Among 3,199 stillbirths, the conditions most commonly identified anywhere in the causal chain were perinatal asphyxia or hypoxia (79.1%), followed by congenital birth defects (9.0%) (Table 2) (Supplementary Table 2). Infectious causes were less common; congenital infections (2.9%) and syphilis (0.8%) were concentrated in South Africa and Mozambique. Cause-of-death distributions varied by site and country: asphyxia or hypoxia accounted for >85% in Bangladesh, Kenya, and Sierra Leone, versus 74.1% in Ethiopia and 60.7% in South Africa (Supplementary Figure 1). The proportion of stillbirths with congenital birth defects was highest in Ethiopia (23.7%), primarily reflecting neural tube defects, including anencephaly or craniorachischisis, spina bifida, encephalocele, and congenital hydrocephalus.

**TABLE 2:** Most common causes of death anywhere in the causal chain* among stillbirths, neonates, and infants and children^†^ in Africa and South Asia — Child Health and Mortality Prevention Surveillance network, seven countries,^§^ 2016–2024

Cause of death	Stillbirth	Neonate^†^	Infant or child^†^	Total
	No. (%)	No. (%)	No. (%)	No. (%)
Perinatal asphyxia or hypoxia	2,529 (79.1)	1,218 (37.7)	4 (0.2)	**3,751 (44.1)**
Sepsis	147 (4.6)	1,178 (36.5)	765 (36.9)	**2,090 (24.6)**
Neonatal preterm birth complication	21 (0.7)	1,281 (39.7)	84 (4.1)	**1,386 (16.3)**
Lower respiratory infection	2 (0.1)	388 (12.0)	775 (37.4)	**1,165 (13.7)**
Congenital birth defect	289 (9.0)	249 (7.7)	166 (8.0)	**704 (8.3)**
Malnutrition	0 (—)	9 (0.3)	566 (27.3)	**575 (6.8)**
Malaria	0 (—)	2 (0.1)	458 (22.1)	**460 (5.4)**
Meningitis or encephalitis	5 (0.2)	249 (7.7)	154 (7.4)	**408 (4.8)**
Diarrheal disease	0 (—)	3 (0.1)	359 (17.3)	**362 (4.3)**
Other neonatal disorder	78 (2.4)	246 (7.6)	37 (1.8)	**361 (4.2)**
Congenital infection	94 (2.9)	184 (5.7)	12 (0.6)	**290 (3.4)**
Anemia	0 (—)	14 (0.4)	234 (11.3)	**248 (2.9)**
Neonatal aspiration syndrome	5 (0.2)	170 (5.3)	0 (—)	**175 (2.1)**
Other infection	8 (0.3)	34 (1.1)	125 (6.0)	**167 (2.0)**
Other respiratory disease	0 (—)	28 (0.9)	136 (6.6)	**164 (1.9)**
HIV infection	0 (—)	3 (0.1)	159 (7.7)	**162 (1.9)**
Other endocrine, metabolic, blood, or immune disorder	0 (—)	15 (0.5)	115 (5.6)	**130 (1.5)**
Neonatal encephalopathy	0 (—)	120 (3.7)	3 (0.1)	**123 (1.4)**
Other	3 (0.1)	34 (1.1)	53 (2.6)	**90 (1.1)**
Injury	0 (—)	3 (0.1)	84 (4.1)	**87 (1.0)**
Other neurologic disorder	0 (—)	14 (0.4)	33 (1.6)	**47 (0.6)**
Syphilis	27 (0.8)	13 (0.4)	4 (0.2)	**44 (0.5)**
Liver disease	0 (—)	7 (0.2)	21 (1.0)	**28 (0.3)**
Sickle cell disorder	0 (—)	0 (—)	28 (1.4)	**28 (0.3)**
Poisoning	1 (<0.1)	0 (—)	23 (1.1)	**24 (0.3)**
Tuberculosis	0 (—)	0 (—)	23 (1.1)	**23 (0.3)**
Kidney disease	1 (<0.1)	11 (0.3)	9 (0.4)	**21 (0.2)**
Heart disease	0 (—)	1 (<0.1)	16 (0.8)	**17 (0.2)**
Placental complication	13 (0.4)	4 (0.1)	0 (—)	**17 (0.2)**
Cancer	1 (<0.1)	0 (—)	14 (0.7)	**15 (0.2)**
Measles	0 (—)	1 (<0.1)	13 (0.6)	**14 (0.2)**
Other nutritional deficiency	0 (—)	0 (—)	14 (0.7)	**14 (0.2)**
Other disorder of fluid, electrolyte, or acid-base balance	0 (—)	3 (0.1)	10 (0.5)	**13 (0.2)**
Paralytic ileus and intestinal obstruction	0 (—)	2 (0.1)	11 (0.5)	**13 (0.2)**
Other immunodeficiency	0 (—)	0 (—)	12 (0.6)	**12 (0.1)**
**Total deaths evaluated**	**3,199 (100)**	**3,230 (100)**	**2,071 (100)**	**8,500 (100)**

* All causes of death implicated in ≥10 deaths across all sites are shown by age group. Deaths could have multiple causes in the causal chain, so the sum of causes is greater than the total number of deaths.

^†^ Neonates were aged ≤27 days, infants were aged 28 days to <12 months, and children were aged 12 months to <60 months.

^§^ Bangladesh, Ethiopia, Kenya, Mali, Mozambique, Sierra Leone, and South Africa.

Among 3,230 neonatal deaths, leading causes identified anywhere in the causal chain were preterm birth complications (39.7%), perinatal asphyxia or hypoxia (37.7%), and sepsis (36.5%); lower respiratory infections (12.0%), meningitis or encephalitis (7.7%), congenital birth defects (7.7%), and congenital infections (5.7%) were also common (Table 2). Preterm birth complications (34.7%) and asphyxia or hypoxia (32.4%) were the most common underlying causes, whereas sepsis (27.0%) and lower respiratory infections (9.4%) more often appeared as antecedent or immediate causes (Supplementary Table 2). The proportion of neonatal deaths with sepsis included anywhere in the causal chain varied across sites (ranging from 21.6% in Kenya to 62.9% in Ethiopia) (Supplementary Figure 1). Preterm birth complications were most frequent in South Africa (60.7%) and Bangladesh (53.2%); perinatal asphyxia or hypoxia in Sierra Leone (56.1%) and Ethiopia (53.0%); congenital birth defects in Mali (16.1%) and South Africa (11.7%); and aspiration syndromes in Ethiopia (15.4%). Across all age groups, when congenital birth defects were present in the causal chain, they were typically assigned as the underlying cause (overall, 668 of 704 [94.9%]).

**FIGURE 3:**
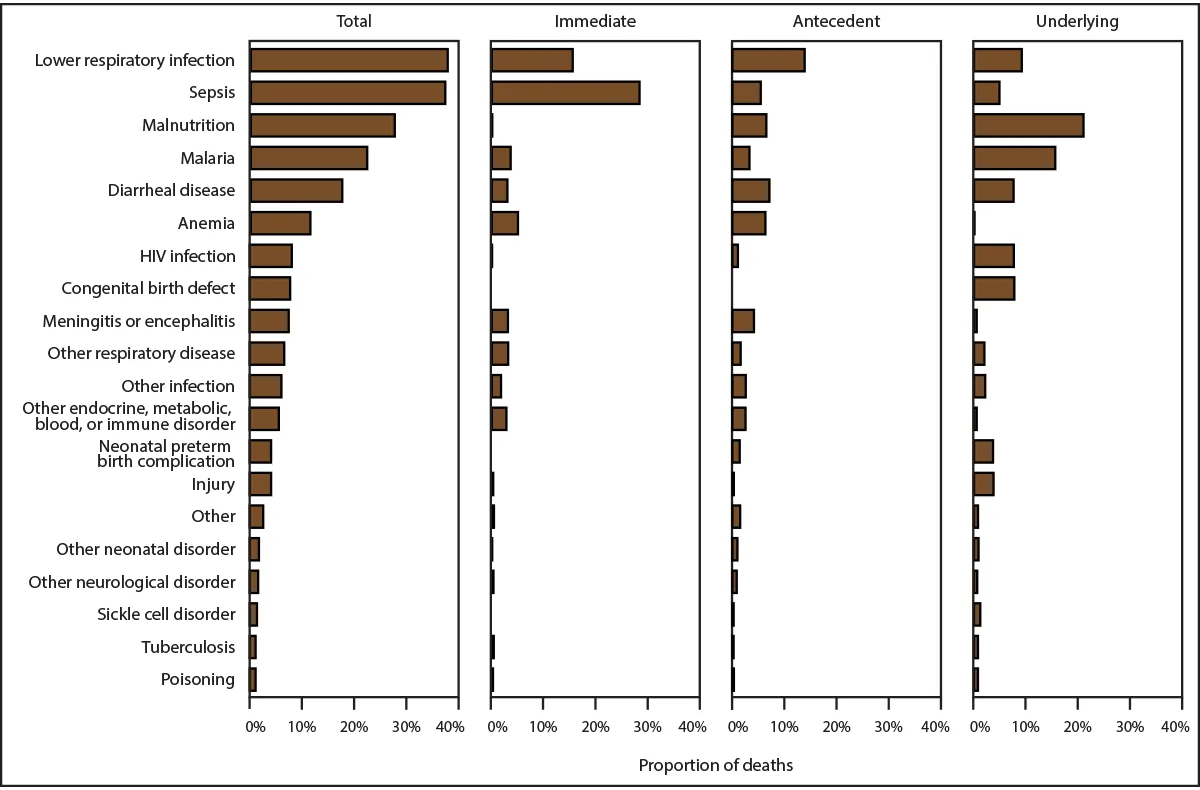
Main causes of death* among infants and children aged <5 years in Africa and South Asia, by position in the causal chain — Child Health and Mortality Prevention Surveillance network, seven countries,^†^ 2016–2024 * N = 2,071 deaths; categories are not mutually exclusive because a death can include multiple causes. ^†^ Bangladesh, Ethiopia, Kenya, Mali, Mozambique, Sierra Leone, and South Africa.

Among 2,071 infant and child deaths, leading conditions identified anywhere in the causal chain of death included lower respiratory infection (37.4%), sepsis (36.9%), malnutrition (27.3%), malaria (22.1%), diarrheal disease (17.3%), anemia (11.3%), congenital birth defects (8.0%), HIV infection (7.7%), and meningitis or encephalitis (7.4%) (Table 2). The most frequent underlying causes were malnutrition (20.8%), malaria (15.5%), and lower respiratory infection (9.1%), whereas sepsis (32.4%), lower respiratory infection (28.8%), and diarrheal disease (9.9%) more often occurred as antecedent or immediate causes (Figure 3) (Supplementary Table 2). Malaria frequently contributed to deaths in Sierra Leone (40.1%), Kenya (31.5%), and Mozambique (20.1%). In Ethiopia, sepsis (72.5%), malnutrition (76.1%), and diarrheal disease (45.8%) each contributed to a substantial proportion of deaths (Supplementary Figure 1).

### Pathogens Identified in the Causal Chain of Death

Among 3,199 stillbirths, a total of 230 (7.2%) had at least one pathogen included in the causal chain of death, most often a single organism (182 of 230 [79.1%]; mean = 1.3 pathogens per death). Gram-negative bacteria (131 of 230 [57.0%]) and gram-positive bacteria (108 of 230 [47.0%]) were the pathogen groups most often included in the causal chain; percentages exceed 100% because certain deaths involved multiple pathogens. The pathogens most often included in the causal chain were *E. coli/Shigella* spp. (65 of 230 [28.3%]), *Streptococcus agalactiae* (54 of 230 [23.5%]), *Treponema pallidum* (29 of 230 [12.6%]), and *Klebsiella pneumoniae* (22 of 230 [9.6%]) (Table 3) (Supplementary Table 3) (Supplementary Figure 2). Among stillbirths with a pathogen included in the causal chain, the proportion with *S. agalactiae* infection varied by site, ranging from 18.2% in Mozambique (four of 22) and 18.4% in Ethiopia (nine of 49) to 33.0% in South Africa (34 of 103). Viral causes were uncommon; cytomegalovirus (24 of 230 [10.4%]) was the virus most often included in the causal chain, and no fungal pathogens were included.

**TABLE 3:** Pathogens identified as causing infectious disease deaths*^,†^ for those deaths associated with one or more pathogens in the causal chain among stillbirths, neonates, and infants and children^§^ in Africa and South Asia — Child Health and Mortality Prevention Surveillance network, seven countries,^¶^ 2016–2024

Pathogen	Stillbirth	Neonate^§^	Infant or child^§^	Total
	No. (%)	No. (%)	No. (%)	No. (%)
**Gram-negative bacteria**	131 (57.0)	1,057 (85.7)	831 (54.9)	**2,019 (67.8)**
*Klebsiella pneumoniae*	22 (9.6)	596 (48.3)	474 (31.3)	**1,092 (36.7)**
*Escherichia coli/Shigella* spp.**	65 (28.3)	179 (14.5)	237 (15.6)	**481 (16.1)**
*Acinetobacter baumannii*	0 (—)	319 (25.9)	92 (6.1)	**411 (13.8)**
*Pseudomonas aeruginosa*	1 (0.4)	84 (6.8)	83 (5.5)	**168 (5.6)**
*Haemophilus influenzae* (HIAT detected; Hib target not detected)^††^	5 (2.2)	12 (1.0)	128 (8.4)	**145 (4.9)**
*Salmonella* spp.	1 (0.4)	30 (2.4)	40 (2.6)	**71 (2.4)**
*Enterobacter cloacae*	4 (1.7)	35 (2.8)	17 (1.1)	**56 (1.9)**
*Moraxella catarrhalis*	0 (—)	7 (0.6)	48 (3.2)	**55 (1.8)**
*Treponema pallidum*	29 (12.6)	14 (1.1)	4 (0.3)	**47 (1.6)**
*Ureaplasma* spp.	13 (5.7)	27 (2.2)	2 (0.1)	**42 (1.4)**
*Serratia marcescens*	0 (—)	36 (2.9)	4 (0.3)	**40 (1.3)**
*Pantoea* spp.	0 (—)	27 (2.2)	1 (0.1)	**28 (0.9)**
*Haemophilus influenzae* (Hib target detected by TAC [HITB])^††^	0 (—)	0 (—)	20 (1.3)	**20 (0.7)**
*Klebsiella* spp.	0 (—)	7 (0.6)	8 (0.5)	**15 (0.5)**
*Neisseria meningitidis*	0 (—)	4 (0.3)	8 (0.5)	**12 (0.4)**
*Campylobacter jejuni*	0 (—)	0 (—)	11 (0.7)	**11 (0.4)**
*Aeromonas* spp.	0 (—)	3 (0.2)	7 (0.5)	**10 (0.3)**
*Bordetella pertussis*	0 (—)	2 (0.2)	8 (0.5)	**10 (0.3)**
*Shigella* spp.	0 (—)	2 (0.2)	8 (0.5)	**10 (0.3)**
*Klebsiella oxytoca*	1 (0.4)	4 (0.3)	4 (0.3)	**9 (0.3)**
*Bordetella* spp.	0 (—)	2 (0.2)	6 (0.4)	**8 (0.3)**
*Burkholderia cepacia*	0 (—)	6 (0.5)	2 (0.1)	**8 (0.3)**
*Proteus mirabilis*	0 (—)	4 (0.3)	4 (0.3)	**8 (0.3)**
*Vibrio cholerae*	0 (—)	2 (0.2)	6 (0.4)	**8 (0.3)**
*Citrobacter freundii*	0 (—)	2 (0.2)	4 (0.3)	**6 (0.2)**
*Morganella morganii*	0 (—)	2 (0.2)	3 (0.2)	**5 (0.2)**
*Serratia liquefaciens*	0 (—)	5 (0.4)	0 (—)	**5 (0.2)**
Other^§§^	5 (2.2)	29 (2.4)	24 (1.6)	**58 (1.9)**
**Gram-positive bacteria**	108 (47.0)	285 (23.1)	522 (34.5)	**915 (30.7)**
*Streptococcus pneumoniae*	5 (2.2)	37 (3.0)	305 (20.1)	**347 (11.6)**
*Staphylococcus aureus*	3 (1.3)	52 (4.2)	95 (6.3)	**150 (5.0)**
*Streptococcus agalactiae*	54 (23.5)	69 (5.6)	10 (0.7)	**133 (4.5)**
*Streptococcus* spp.	15 (6.5)	31 (2.5)	50 (3.3)	**96 (3.2)**
*Enterococcus faecalis*	23 (10.0)	34 (2.8)	32 (2.1)	**89 (3.0)**
*Enterococcus faecium*	1 (0.4)	35 (2.8)	36 (2.4)	**72 (2.4)**
*Streptococcus pyogenes*	2 (0.9)	12 (1.0)	25 (1.7)	**39 (1.3)**
*Mycobacterium tuberculosis*	0 (—)	0 (—)	22 (1.5)	**22 (0.7)**
*Enterococcus* spp.	5 (2.2)	8 (0.6)	5 (0.3)	**18 (0.6)**
Coagulase-negative *Staphylococcus*	3 (1.3)	6 (0.5)	2 (0.1)	**11 (0.4)**
*Staphylococcus* spp.	0 (—)	7 (0.6)	4 (0.3)	**11 (0.4)**
*Listeria monocytogenes*	1 (0.4)	8 (0.6)	0 (—)	**9 (0.3)**
*Viridans streptococcus*	1 (0.4)	2 (0.2)	6 (0.4)	**9 (0.3)**
Other^§§^	2 (0.9)	9 (0.7)	5 (0.3)	**16 (0.5)**
**Viruses**	28 (12.2)	59 (4.8)	480 (31.7)	**567 (19.0)**
Cytomegalovirus	24 (10.4)	21 (1.7)	129 (8.5)	**174 (5.8)**
HIV	0 (—)	3 (0.2)	155 (10.2)	**158 (5.3)**
Adenovirus	1 (0.4)	0 (—)	89 (5.9)	**90 (3.0)**
Respiratory syncytial virus	0 (—)	13 (1.1)	43 (2.8)	**56 (1.9)**
Rotavirus A	0 (—)	0 (—)	28 (1.8)	**28 (0.9)**
Rhinovirus	0 (—)	1 (0.1)	18 (1.2)	**19 (0.6)**
Parainfluenza virus type 3	0 (—)	2 (0.2)	14 (0.9)	**16 (0.5)**
Measles	1 (0.4)	1 (0.1)	13 (0.9)	**15 (0.5)**
Rotavirus, nontypeable	0 (—)	0 (—)	15 (1.0)	**15 (0.5)**
Influenza A	0 (—)	0 (—)	12 (0.8)	**12 (0.4)**
SARS-CoV-2	0 (—)	6 (0.5)	6 (0.4)	**12 (0.4)**
Parvovirus B19	1 (0.4)	2 (0.2)	8 (0.5)	**11 (0.4)**
Enterovirus	0 (—)	2 (0.2)	7 (0.5)	**9 (0.3)**
Human metapneumovirus	0 (—)	1 (0.1)	8 (0.5)	**9 (0.3)**
Norovirus genogroup GII	0 (—)	0 (—)	9 (0.6)	**9 (0.3)**
Influenza B	0 (—)	1 (0.1)	6 (0.4)	**7 (0.2)**
Parainfluenza virus type 1	0 (—)	0 (—)	7 (0.5)	**7 (0.2)**
Norovirus genogroup GI	0 (—)	0 (—)	6 (0.4)	**6 (0.2)**
Other^§§^	1 (0.4)	7 (0.6)	16 (1.1)	**24 (0.8)**
**Fungi**	0 (—)	68 (5.5)	108 (7.1)	**176 (5.9)**
*Candida albicans*	0 (—)	34 (2.8)	28 (1.8)	**62 (2.1)**
*Pneumocystis jirovecii*	0 (—)	0 (—)	52 (3.4)	**52 (1.7)**
*Candida* spp.	0 (—)	11 (0.9)	16 (1.1)	**27 (0.9)**
*Candida parapsilosis*	0 (—)	6 (0.5)	6 (0.4)	**12 (0.4)**
*Candida auris*	0 (—)	6 (0.5)	4 (0.3)	**10 (0.3)**
*Candida glabrata*	0 (—)	7 (0.6)	0 (—)	**7 (0.2)**
Other^§§^	0 (—)	7 (0.6)	4 (0.3)	**11 (0.4)**
**Parasites**	1 (0.4)	8 (0.6)	420 (27.7)	**429 (14.4)**
*Plasmodium falciparum*	1 (0.4)	5 (0.4)	408 (26.9)	**414 (13.9)**
Other^§§^	0 (—)	3 (0.2)	12 (0.8)	**15 (0.5)**
**Total number of infectious disease deaths with a pathogen identified**	**230 (100)**	**1,234 (100)**	**1,515 (100)**	**2,979 (100)**

**Abbreviations:** CHAMPS = Child Health and Mortality Prevention Surveillance; HIAT = pan-genome *Haemophilus influenzae* detection assay; Hib = *H. influenzae* type b; HITB = Hib target assay; TAC = TaqMan array card.

* Infectious disease deaths were defined as deaths in which an infectious cause was included in the causal chain. Causes of death included sepsis, meningitis or encephalitis, lower respiratory infection, upper respiratory infection, diarrheal disease with specific pathogen identified, malaria, HIV, measles, rabies, syphilis, tuberculosis, congenital infection, and other specified infection.

^†^ Of 8,500 deaths (3,199 stillbirths; 3,230 neonates; and 2,071 infants and children) that had completed CHAMPS determination of cause-of-death analysis, a total of 3,415 deaths (269 stillbirths; 1,423 neonatal deaths; and 1,723 infant and child deaths) were determined to be caused by an infectious disease; a pathogen was not identified in all infectious disease deaths. Because certain deaths were associated with multiple pathogens in the causal chain, the sum of all values in each column is greater than the column total.

^§^ Neonates were aged ≤27 days, infants were aged 28 days to <12 months, and children were aged 12 months to <60 months.

^¶^ Bangladesh, Ethiopia, Kenya, Mali, Mozambique, Sierra Leone, and South Africa.

** Because certain TAC targets are shared by *Escherichia* *coli* and *Shigella* spp./enteroinvasive *E. coli*, these detections are reported as “*E. coli*/*Shigella* spp.”

^††^ CHAMPS TAC includes an HIAT that detects encapsulated (types a–f) and nonencapsulated *H. influenzae* strains and an HITB intended for Hib (with rare cross-reactivity with *H. influenzae* type a). HITB positivity does not exclude mixed types; a positive HIAT result without a positive HITB result does not distinguish nonencapsulated from non-Hib encapsulated strains. Therefore, results are reported as “Hib target detected by TAC” or “HIAT detected; Hib target not detected,” rather than as a definitive serotype.

^§§^ Other category includes single pathogens with fewer than five detections per pathogen.

Among 3,230 neonatal deaths, at least one pathogen was included in the causal chain of death for 1,234 (38.2%), with one pathogen in 786 (63.7%), two in 283 (22.9%), and three or more in 165 (13.4%) (mean = 1.5 per death). Gram-negative bacteria were the most frequently identified pathogen group in the causal chain (1,057 of 1,234 [85.7%]), particularly *K. pneumoniae* (596 of 1,234 [48.3%]), *Acinetobacter baumannii* (319 of 1,234 [25.9%]), and *E. coli/Shigella* spp. (179 of 1,234 [14.5%]) (Table 3). These bacteria were typically assigned as antecedent or immediate causes and were most often implicated in sepsis, lower respiratory infection, and meningitis and encephalitis (Supplementary Table 3) (Supplementary Figure 3). Gram-positive bacteria were included in the causal chain in 285 (23.1%) deaths, most often *S. agalactiae* (69 of 1,234 [5.6%]), *Staphylococcus aureus* (52 of 1,234 [4.2%]), and *Streptococcus pneumoniae* (37 of 1,234 [3.0%]) (Table 3). Among neonatal deaths with a pathogen included in the causal chain, the proportion with *S. agalactiae* varied by site, ranging from 5.1% in Mali (seven of 138) and 8.0% in Mozambique (11 of 137) to 11.4% in South Africa (41 of 360). Fungal pathogens were included in the causal chain in 68 (5.5%) deaths, predominantly *Candida albicans* (34 of 1,234 [2.8%]). Viral pathogens were included in the causal chain in 59 (4.8%) deaths, most often cytomegalovirus (21 of 1,234 [1.7%]). Across sites, *K. pneumoniae* was the pathogen most frequently included in the causal chain, whereas *A. baumannii* was disproportionately frequent in sites in South Africa (207 of 360 [57.5%]) and Bangladesh (67 of 165 [40.6%]) (Supplementary Figure 4).

Among 2,071 infant and child deaths, at least one pathogen was included in the causal chain for 1,515 (73.2%). Among these 1,515 deaths, the mean number of pathogens included in the causal chain was 2.0 per death (range = 1–7): one pathogen was identified in 739 (48.8%) deaths, two in 362 (23.9%), and three or more in 414 (27.3%). Most infant and child deaths with a pathogen included in the causal chain occurred in health care facilities (1,075 of 1,515 [71.0%]). Gram-negative organisms were included in the causal chain in 831 (54.9%) deaths, most often *K. pneumoniae* (474 of 1,515 [31.3%]), *E. coli/Shigella* spp. (237 of 1,515 [15.6%]), and *Haemophilus influenzae* (detected by a pangenome *H.* *influenzae* detection assay; *H.* *influenzae* type b [Hib] target not detected) (128 of 1,515 [8.4%]). Gram-positive bacteria were included in the causal chain in 522 (34.5%) deaths, most often *S. pneumoniae* (305 of 1,515 [20.1%]) and *S. aureus* (95 of 1,515 [6.3%]) (Table 3) (Supplementary Figures 5 and 6). Viral pathogens were included in the causal chain in 480 (31.7%) deaths, most commonly cytomegalovirus (129 of 1,515 [8.5%]) and adenovirus (89 of 1,515 [5.9%]); parasitic pathogens were included in 420 (27.7%) deaths, primarily *Plasmodium falciparum* (408 of 1,515 [26.9%]); and fungal pathogens were included in 108 (7.1%) deaths, including *Pneumocystis jirovecii* (52 of 1,515 [3.4%]) and *C. albicans* (28 of 1,515 [1.8%]). Among 408 deaths with *P. falciparum* in the causal chain, 92 (22.5%) also had at least one bacterial pathogen in the causal chain. Among infant and child deaths with a pathogen included in the causal chain, the proportion with *S. pneumoniae* was highest in Ethiopia (60 of 125 [48.0%]), Mali (50 of 134 [37.3%]), and Mozambique (62 of 195 [31.8%]).

### Maternal Conditions Associated with Stillbirths and Neonatal Deaths

A main maternal condition was assigned for 2,273 of 3,199 stillbirths (71.1%) and 1,879 of 3,230 neonatal deaths (58.2%) (Table 4) (Supplementary Tables 4 and 5). Among stillbirths, the most frequent *ICD-PM* maternal condition groups were M1, complications of the placenta, cord, or membranes (891 of 3,199 [27.9%]); M4, maternal medical or surgical conditions (748 of 3,199 [23.4%]); M3, other complications of labor and delivery (345 of 3,199 [10.8%]); and M2, maternal complications of pregnancy (168 of 3,199 [5.3%]). Among neonatal deaths, the most frequent maternal condition groups were M4 (484 of 3,230 [15.0%]), M3 (468 of 3,230 [14.5%]), M2 (455 of 3,230 [14.1%]), and M1 (309 of 3,230 [9.6%]).

**TABLE 4:** Underlying causes of stillbirths and neonatal deaths in Africa and South Asia, by main maternal condition group* — Child Health and Mortality Prevention Surveillance network, seven countries,^†^ 2016–2024

Underlying cause of stillbirth or neonatal death	Maternal condition *ICD-PM* codes					Other	Total
	M1: placenta, cord or membrane complications	M2: maternal complications of pregnancy	M3: other complications of labor and delivery	M4: maternal medical or surgical conditions	M5: no condition identified		
	No. (%)	No. (%)	No. (%)	No. (%)	No. (%)	No. (%)	No.
Perinatal asphyxia or hypoxia	853 (71.1)	241 (38.7)	602 (74.0)	785 (63.7)	797 (35.0)	144 (50.7)	**3,422**
Neonatal preterm birth complication	142 (11.8)	264 (42.4)	123 (15.1)	251 (20.4)	282 (12.4)	65 (22.9)	**1,127**
Congenital birth defect	9 (0.8)	10 (1.6)	15 (1.8)	18 (1.5)	437 (19.2)	22 (7.7)	**511**
Sepsis	90 (7.5)	52 (8.3)	17 (2.1)	45 (3.7)	224 (9.8)	13 (4.6)	**441**
Undetermined	11 (0.9)	7 (1.1)	1 (0.1)	12 (1.0)	224 (9.8)	5 (1.8)	**260**
Congenital infection	43 (3.6)	14 (2.2)	11 (1.4)	35 (2.8)	76 (3.3)	7 (2.5)	**186**
Other neonatal disorder	20 (1.7)	10 (1.6)	4 (0.5)	26 (2.1)	46 (2.0)	8 (2.8)	**114**
Lower respiratory infection	4 (0.3)	7 (1.1)	7 (0.9)	5 (0.4)	60 (2.6)	3 (1.1)	**86**
Neonatal aspiration syndrome	4 (0.3)	8 (1.3)	18 (2.2)	6 (0.5)	38 (1.7)	8 (2.8)	**82**
Syphilis	6 (0.5)	0 (—)	0 (—)	27 (2.2)	7 (0.3)	0 (—)	**40**
Neonatal encephalopathy	3 (0.2)	1 (0.2)	8 (1.0)	3 (0.2)	10 (0.4)	4 (1.4)	**29**
Other	3 (0.2)	1 (0.2)	1 (0.1)	2 (0.2)	14 (0.6)	2 (0.7)	**23**
Other infection	2 (0.2)	1 (0.2)	2 (0.2)	5 (0.4)	12 (0.5)	0 (—)	**22**
Meningitis or encephalitis	0 (—)	1 (0.2)	0 (—)	3 (0.2)	10 (0.4)	0 (—)	**14**
Placental complication	3 (0.2)	1 (0.2)	0 (—)	3 (0.2)	7 (0.3)	0 (—)	**14**
Chorioamnionitis and membrane complication	3 (0.2)	2 (0.3)	0 (—)	0 (—)	3 (0.1)	0 (—)	**8**
Other maternal factor	1 (0.1)	0 (—)	0 (—)	3 (0.2)	2 (0.1)	1 (0.4)	**7**
Umbilical cord complication	3 (0.2)	0 (—)	0 (—)	0 (—)	3 (0.1)	1 (0.4)	**7**
Malnutrition	0 (—)	0 (—)	0 (—)	0 (—)	5 (0.2)	1 (0.4)	**6**
Other respiratory disease	0 (—)	1 (0.2)	0 (—)	0 (—)	4 (0.2)	0 (—)	**5**
Obstructed labor and fetal malpresentation	0 (—)	0 (—)	3 (0.4)	0 (—)	1 (<0.1)	0 (—)	**4**
HIV infection	0 (—)	0 (—)	0 (—)	2 (0.2)	1 (<0.1)	0 (—)	**3**
Liver disease	0 (—)	0 (—)	0 (—)	0 (—)	3 (0.1)	0 (—)	**3**
Other endocrine, metabolic, blood, or immune disorder	0 (—)	1 (0.2)	0 (—)	0 (—)	2 (0.1)	0 (—)	**3**
Birth trauma	0 (—)	0 (—)	1 (0.1)	0 (—)	1 (<0.1)	0 (—)	**2**
Diarrheal disease	0 (—)	0 (—)	0 (—)	0 (—)	2 (0.1)	0 (—)	**2**
Injury	0 (—)	0 (—)	0 (—)	0 (—)	2 (0.1)	0 (—)	**2**
Kidney disease	0 (—)	0 (—)	0 (—)	0 (—)	2 (0.1)	0 (—)	**2**
Other labor and delivery complication	0 (—)	1 (0.2)	0 (—)	1 (0.1)	0 (—)	0 (—)	**2**
Other neurologic disorder	0 (—)	0 (—)	0 (—)	0 (—)	2 (0.1)	0 (—)	**2**
**Total**	**1,200 (100)**	**623 (100)**	**813 (100)**	**1,232 (100)**	**2,277 (100)**	**284 (100)**	**6,429**

**Abbreviation:** *ICD-PM =* *International Classification of Diseases for Perinatal Mortality.*

* Each death was assigned one main maternal condition in the causal chain, as determined by the Child Health and Mortality Prevention Surveillance determination of cause-of-death panel. Percentages are column percentages; within each maternal condition code group, they represent the proportion of deaths assigned to the specified underlying cause. Percentages therefore sum to 100% within columns, allowing for rounding.

^†^ Bangladesh, Ethiopia, Kenya, Mali, Mozambique, Sierra Leone, and South Africa.

Among stillbirths, the most frequent main maternal conditions were placental complications (539 of 3,199 [16.8%]), primarily placental abruption or other placental separation with hemorrhage, and hypertensive disorders of pregnancy (537 of 3,199 [16.8%]). Hypertensive disorders were most frequent in South Africa (102 of 374 [27.3%]), Kenya (72 of 381 [18.9%]), and Bangladesh (98 of 543 [18.0%]) (Supplementary Table 4). Placental complications were most frequent in Mali (92 of 276 [33.3%]), South Africa (80 of 374 [21.4%]), and Sierra Leone (61 of 303 [20.1%]). Chorioamnionitis and membrane complications accounted for 6.9% of stillbirths (220 of 3,199) and umbilical cord complications for 3.5% (112 of 3,199).

Among neonatal deaths, the most frequent main maternal conditions were hypertensive disorders of pregnancy (294 of 3,230 [9.1%]), often linked to asphyxia or hypoxia, followed by multiple gestation (227 of 3,230 [7.0%]), premature rupture of membranes (171 of 3,230 [5.3%]), and preterm labor or delivery (117 of 3,230 [3.6%]) (Supplementary Table 5). The proportion of neonatal deaths with placenta, cord, or membrane complications (group M1) as the assigned maternal condition was highest in South Africa (89 of 616 [14.4%]), Sierra Leone (39 of 374 [10.4%]), Mozambique (67 of 655 [10.2%]), and Mali (31 of 305 [10.2%]) (Supplementary Table 5).

Across stillbirths and neonatal deaths, perinatal asphyxia or hypoxia was the most common underlying cause among deaths with placenta, cord, or membrane complications (group M1; 853 of 1,200 [71.1%]) and other labor and delivery complications (group M3; 602 of 813 [74.0%]) (Table 4). Preterm birth complications were the leading underlying cause among deaths with maternal complications of pregnancy (group M2; 264 of 623 [42.4%]). Among deaths with no main maternal condition identified (group M5), congenital birth defects accounted for 19.2% (437 of 2,277).

### Preventability of Deaths

Among 8,500 deaths analyzed, 7,558 (88.9%) had an overall preventability classification; among these, 6,103 (80.7%) were classified as preventable (n = 5,323) or as possibly preventable under certain circumstances (n = 780). Specific health care system improvement recommendations were documented for 6,276 deaths and are summarized overall (Table 5) and by site and age group (Supplementary Figure 7). The most frequently identified opportunities for improvement were in antenatal care (2,684 of 6,276 [42.8%]), obstetric care and management (2,460 of 6,276 [39.2%]), health care–seeking behavior (2,285 of 6,276 [36.4%]), and pediatric clinical management and quality of care (2,094 of 6,276 [33.4%]). Additional gaps included health education (1,563 of 6,276 [24.9%]), infection prevention and control (955 of 6,276 [15.2%]), nutritional support (653 of 6,276 [10.4%]), preconception counseling and family planning (566 of 6,276 [9.0%]), HIV infection prevention and control (225 of 6,276 [3.6%]), and transport systems (225 of 6,276 [3.6%]). These recommendations often reflected gaps in diagnostic capacity, timely referral, and access to recommended treatment, particularly for severe infections.

**TABLE 5:** Health care system improvement recommendations for stillbirths and neonatal and infant and child deaths*^,†^ in Africa and South Asia, by recommendation category and age group — Child Health and Mortality Prevention Surveillance network, seven countries,^§^ 2016–2024

Health care system improvement recommendation category	Stillbirth	Neonate^†^	Infant or child^†^	Total
	No. (%)	No. (%)	No. (%)	No. (%)
**Antenatal care**	1,542 (68.7)	1,087 (44.7)	55 (3.4)	**2,684 (42.8)**
Clinical management	974 (43.4)	588 (24.2)	32 (2.0)	**1,594 (25.4)**
Health screening and diagnosis	907 (40.4)	547 (22.5)	27 (1.7)	**1,481 (23.6)**
Quality antenatal care	776 (34.6)	603 (24.8)	33 (2.1)	**1,412 (22.5)**
Fetal screening and growth monitoring	648 (28.9)	378 (15.5)	10 (0.6)	**1,036 (16.5)**
Health assessment	629 (28.0)	377 (15.5)	24 (1.5)	**1,030 (16.4)**
Referral to specialty care	656 (29.2)	351 (14.4)	18 (1.1)	**1,025 (16.3)**
Preparation and planning for safe delivery	593 (26.4)	378 (15.5)	13 (0.8)	**984 (15.7)**
**Obstetric care and management**	1,246 (55.5)	1,185 (48.7)	29 (1.8)	**2,460 (39.2)**
Clinical management of complications	798 (35.6)	706 (29.0)	8 (0.5)	**1,512 (24.1)**
Monitoring progress and fetus during labor and delivery	769 (34.3)	717 (29.5)	10 (0.6)	**1,496 (23.8)**
Health assessment during labor and delivery	633 (28.2)	590 (24.3)	13 (0.8)	**1,236 (19.7)**
Health screening and diagnosis during labor and delivery	595 (26.5)	532 (21.9)	16 (1.0)	**1,143 (18.2)**
Access to qualified health care provider for delivery	451 (20.1)	498 (20.5)	16 (1.0)	**965 (15.4)**
Access to emergency obstetric care	438 (19.5)	359 (14.8)	7 (0.4)	**804 (12.8)**
Referral during labor and delivery for advanced care	392 (17.5)	340 (14.0)	3 (0.2)	**735 (11.7)**
Documentation of maternal medical records from labor and delivery	278 (12.4)	258 (10.6)	7 (0.4)	**543 (8.7)**
**Health care–seeking behavior**	900 (40.1)	612 (25.2)	773 (48.3)	**2,285 (36.4)**
Response to condition or emergency	702 (31.3)	439 (18.1)	584 (36.5)	**1,725 (27.5)**
Prevention	616 (27.5)	349 (14.4)	454 (28.4)	**1,419 (22.6)**
Family adherence to medical advice and use of appropriate treatments	221 (9.8)	157 (6.5)	325 (20.3)	**703 (11.2)**
**Pediatric clinical management and quality of care**	88 (3.9)	1,086 (44.7)	920 (57.5)	**2,094 (33.4)**
Clinical management of pediatric care	22 (1.0)	546 (22.5)	702 (43.8)	**1,270 (20.2)**
Pediatric health screening and diagnosis	20 (0.9)	220 (9.0)	449 (28.0)	**689 (11.0)**
Access to newborn intensive care	67 (3.0)	567 (23.3)	16 (1.0)	**650 (10.4)**
Pediatric health assessment	19 (0.8)	225 (9.3)	403 (25.2)	**647 (10.3)**
Access to quality pediatric health care	11 (0.5)	234 (9.6)	349 (21.8)	**594 (9.5)**
Referral for advanced pediatric care	13 (0.6)	149 (6.1)	321 (20.0)	**483 (7.7)**
Newborn monitoring at health care facility	30 (1.3)	346 (14.2)	91 (5.7)	**467 (7.4)**
Access to emergency pediatric care	19 (0.8)	175 (7.2)	128 (8.0)	**322 (5.1)**
Documentation of child medical records	7 (0.3)	124 (5.1)	163 (10.2)	**294 (4.7)**
Documentation of maternal medical history relevant to pediatric health	14 (0.6)	105 (4.3)	39 (2.4)	**158 (2.5)**
**Health education (e.g., vaccinations and preventing malnutrition, diarrhea, burns, and poisoning)**	536 (23.9)	341 (14.0)	686 (42.8)	**1,563 (24.9)**
Newborn and child health	131 (5.8)	147 (6.0)	656 (41.0)	**934 (14.9)**
Pregnancy	507 (22.6)	266 (10.9)	80 (5.0)	**853 (13.6)**
**Infection prevention and control**	34 (1.5)	603 (24.8)	318 (19.9)	**955 (15.2)**
Access to infection prevention and control equipment, supplies, and consumables	19 (0.8)	494 (20.3)	241 (15.1)	**754 (12.0)**
Staff capacity	12 (0.5)	430 (17.7)	191 (11.9)	**633 (10.1)**
Institutional protocols	18 (0.8)	257 (10.6)	123 (7.7)	**398 (6.3)**
**Nutritional support**	174 (7.8)	57 (2.3)	422 (26.4)	**653 (10.4)**
Home malnutrition management for child	3 (0.1)	16 (0.7)	397 (24.8)	**416 (6.6)**
Micronutrient supplementation during pregnancy	161 (7.2)	26 (1.1)	24 (1.5)	**211 (3.4)**
Maternal nutrition support	131 (5.8)	26 (1.1)	15 (0.9)	**172 (2.7)**
**Preconception counseling and family planning**	336 (15.0)	205 (8.4)	25 (1.6)	**566 (9.0)**
**HIV infection prevention and control**	34 (1.5)	44 (1.8)	147 (9.2)	**225 (3.6)**
Child	5 (0.2)	23 (0.9)	135 (8.4)	**163 (2.6)**
Mother	32 (1.4)	37 (1.5)	68 (4.2)	**137 (2.2)**
**Transportation system**	98 (4.4)	87 (3.6)	40 (2.5)	**225 (3.6)**
Access to safe and timely transportation to health care facility	71 (3.2)	52 (2.1)	31 (1.9)	**154 (2.5)**
Access to ambulance	64 (2.9)	54 (2.2)	12 (0.7)	**130 (2.1)**
**Vaccinations**	5 (0.2)	7 (0.3)	120 (7.5)	**132 (2.1)**
Child	0 (—)	4 (0.2)	104 (6.5)	**108 (1.7)**
Mother	5 (0.2)	1 (<0.1)	3 (0.2)	**9 (0.1)**
**Social issues**	42 (1.9)	42 (1.7)	45 (2.8)	**129 (2.1)**
Teenage pregnancy	24 (1.1)	23 (0.9)	7 (0.4)	**54 (0.9)**
Child family care and support	3 (0.1)	1 (<0.1)	31 (1.9)	**35 (0.6)**
Maternal family care and support	8 (0.4)	13 (0.5)	11 (0.7)	**32 (0.5)**
Financial support	2 (0.1)	7 (0.3)	18 (1.1)	**27 (0.4)**
Maternal domestic and sexual violence	7 (0.3)	4 (0.2)	1 (0.1)	**12 (0.2)**
**Other**	208 (9.3)	151 (6.2)	134 (8.4)	**493 (7.9)**
**Total**	**2,244 (100)**	**2,431 (100)**	**1,601 (100)**	**6,276 (100)**

* One or more specific health care system improvement recommendations were documented for 6,276 deaths. Percentages were calculated using deaths with one or more documented recommendations within each age group as the denominator. Multiple recommendation categories could be selected for a given death; therefore, percentages might sum to >100% within an age group.

^†^ Neonates were aged ≤27 days, infants were aged 28 days to <12 months, and children were aged 12 months to <60 months.

^§^ Bangladesh, Ethiopia, Kenya, Mali, Mozambique, Sierra Leone, and South Africa.

Prevention opportunities varied by age group. Among stillbirths with recommendations (n = 2,244), recommendations most often focused on antenatal care (1,542 of 2,244 [68.7%]) and obstetric care and management (1,246 of 2,244 [55.5%]) (Table 5), including clinical management of pregnancy complications, screening and diagnosis, and fetal monitoring during labor and delivery. Among neonatal deaths with recommendations (n = 2,431), the most frequently identified priorities were obstetric care and management (1,185 of 2,431 [48.7%]), antenatal care (1,087 of 2,431 [44.7%]), and pediatric clinical management and quality of care (1,086 of 2,431 [44.7%]), reflecting susceptibilities spanning late pregnancy, delivery, and the immediate postnatal period. Infection prevention and control was identified for 24.8% (603 of 2,431) of neonatal deaths, particularly at sites where *K. pneumoniae* and *A. baumannii* were major causes of sepsis. Among infant and child deaths with recommendations (n = 1,601), recommendations most often addressed pediatric clinical management and quality of care (920 of 1,601 [57.5%]), health care–seeking behavior (773 of 1,601 [48.3%]), and health education (686 of 1,601 [42.8%]), as well as gaps in nutritional support (422 of 1,601 [26.4%]) and infection prevention and control (318 of 1,601 [19.9%]).

## Discussion

This analysis of CHAMPS mortality surveillance data from Bangladesh and six African countries during 2016–2024 provides standardized, multisource postmortem evidence on causes and preventability of stillbirths and deaths among neonates, infants, and children aged <5 years in high-mortality settings. Among 8,500 deaths investigated by MITS and reviewed by multidisciplinary DeCoDe panels, infections, intrapartum complications, preterm birth complications, and malnutrition often occurred together within multistep causal chains that also involved maternal conditions, comorbidities, and gaps in care. By integrating histopathology, microbiology, clinical and maternal record review, verbal autopsy, and expert interpretation, CHAMPS complements verbal autopsy, facility records, and routine mortality reporting by identifying not only broad causes of death but also specific pathogens, maternal contributors, and modifiable factors relevant to prevention.

### Overall Mortality Findings

Cause-of-death patterns varied by age group, reflecting transitions in risk from pregnancy and delivery through the neonatal period and early childhood. Most stillbirths were attributed to perinatal asphyxia or hypoxia, often in the context of maternal or placental conditions such as hypertensive disorders of pregnancy, placental complications, and chorioamnionitis. These findings highlight opportunities for improved antenatal detection, intrapartum monitoring, timely referral, and emergency obstetric management (*34*,*35*). Neonatal deaths were primarily attributed to preterm birth complications, perinatal asphyxia or hypoxia, and sepsis. Gram-negative bacteria, especially *K. pneumoniae* and *A. baumannii*, were frequently included in the causal chain of neonatal deaths. Previous analyses of CHAMPS data suggested that many infections involving these pathogens were likely associated with health care, although community acquisition also occurred (especially for *K. pneumoniae*) and the acquisition setting could not be established for every death (*9*,*17*,*36*). These findings reinforce the importance of infection prevention and control, timely recognition and management of neonatal infection, and quality improvement in maternity and newborn health care units (*36*,*37*).

Among infants and children, deaths commonly involved both infectious and nutritional causes, including lower respiratory infections, sepsis, diarrheal disease, and malnutrition. Malaria and HIV infection contributed substantially to the number of deaths at certain sites, indicating persistent gaps in access to, uptake of, or quality of established prevention and treatment services (*31*,*32*). Malnutrition affected approximately half of children with anthropometric measurements and was frequently included in the causal chain, underscoring the close relationship between undernutrition and infectious mortality (*26*,*38*,*39*). These findings support integrated clinical and public health approaches that can identify and manage overlapping risks, including infection, prematurity, growth failure, HIV exposure or infection, and barriers to timely care, rather than single-disease approaches alone (*3*,*13*,*40*).

The frequent identification of multiple conditions in the causal chain is a central finding. Conventional mortality data often assign a single underlying cause, which can obscure preventable contributors such as maternal complications, malnutrition, delayed care-seeking, missed antenatal screening, inadequate intrapartum monitoring, or health care–associated infection risks. CHAMPS provides a more complete picture of causal pathways by distinguishing underlying, antecedent, and immediate causes of death and by evaluating whether pathogens or other findings plausibly contributed to death. This approach is particularly important in high-mortality settings, where children often seek care late with severe illness and multiple coexisting conditions.

### Interpretation in the Context of Global Evidence

Cause-of-death patterns across CHAMPS sites broadly align with global estimates from the United Nations Inter-agency Group for Child Mortality Estimation and WHO, while providing greater etiologic specificity and additional evidence on comorbidities, maternal contributors, and health care system gaps (*1*). The predominance of intrapartum-related stillbirths and early neonatal deaths and the substantial number of deaths attributed to infectious diseases among neonates, infants, and children are consistent with previous studies (*41*,*42*). CHAMPS links these broad categories to specific pathogens and modifiable circumstances. For example, the prominence of *K. pneumoniae* across sites highlights the importance of infection prevention and control, diagnostic capacity, prompt empiric management of suspected severe bacterial infection, and development of preventive tools, including vaccines against major neonatal and pediatric bacterial pathogens (*17*,*36*).

CHAMPS findings also underscore the continuing contribution of maternal hypertensive disorders and infections such as HIV and syphilis to fetal and neonatal deaths (*9*,*25*). Stillbirths and neonatal deaths comprised approximately three fourths of MITS-investigated deaths, emphasizing the concentration of deaths during pregnancy, labor, delivery, and the early newborn period. As deaths among older children decline, preventing fetal and neonatal deaths will require sustained attention to timely and high-quality antenatal, obstetric, and newborn care. These findings are consistent with WHO and UNICEF priorities, including the Every Newborn Action Plan and the Global Strategy for Women’s, Children’s, and Adolescents’ Health, and are most useful when translated into district- and facility-level action through routine dissemination to health authorities, linkage with child and perinatal death review processes, and audit and feedback to address modifiable gaps in care and referral (*43*–*45*).

CHAMPS also demonstrates the value of postmortem surveillance as a complement to routine mortality systems. By operating within defined catchment areas and, in certain sites, linking with public health and demographic surveillance systems, CHAMPS can support interpretation of mortality patterns and comparison with model-based estimates (*23*,*46*). The system also generates evidence that routine data sources often cannot provide, including histopathologic evidence of disease processes, pathogen-specific attribution, maternal contributors to stillbirth and neonatal death, and structured assessments of preventability.

### Preventability and Public Health Implications

Approximately four in five deaths with a preventability assessment were considered preventable or possibly preventable under certain circumstances. This finding does not imply that every death could have been prevented by a single intervention; rather, it indicates that many deaths occurred along pathways in which earlier recognition, timely referral, improved quality of care, or better implementation of existing interventions might plausibly have altered the outcome. The most common opportunities identified by DeCoDe panels included strengthened antenatal care, improved intrapartum and emergency obstetric management, infection prevention and control, improved pediatric case management, nutritional support, and earlier health care–seeking for pregnancy complications and childhood illness.

These priorities are consistent with evidence that expanding access to and improving delivery of established maternal, newborn, and child health interventions can prevent many stillbirths and neonatal and child deaths. Relevant interventions include high-quality antenatal and intrapartum care; screening for and management of hypertensive disorders, diabetes, HIV infection, and syphilis; antenatal corticosteroids when indicated for threatened preterm birth; timely obstetric referral; neonatal resuscitation and supportive care; prevention and treatment of severe bacterial infection; oxygen and other supportive therapies for severe pneumonia; nutritional support; malaria prevention and treatment; prevention of mother-to-child transmission of HIV; and equitable receipt of routine childhood vaccines (*36*,*47*–*53*). CHAMPS also identified neural tube defects as an important contributor to stillbirth and neonatal mortality in Ethiopia, supporting the potential value of periconceptional folic acid intake and food fortification where feasible (*54*).

The high proportion of preventable deaths suggests that the major gaps often are not the absence of known interventions but incomplete implementation, uneven quality, delayed access, shortages of staff or commodities, weak referral systems, and limited accountability (*55*). Social and behavioral findings from CHAMPS have also highlighted barriers to timely care, including distance, cost, mistrust, and reliance on traditional medicine, emphasizing that clinical improvements must be paired with strategies that reduce barriers to access and strengthen community trust (*24*,*56*).

 Across CHAMPS sites, findings have guided preventive and quality-improvement activities. Beginning in 2022, CHAMPS findings informed a package of interventions in Siaya County, Kenya, including expansion of Kangaroo Mother Care and training in emergency obstetric care and neonatal triage, assessment, and treatment. From 2022 to 2023, Kangaroo Mother Care admissions nearly doubled, while crude mortality among combined newborn-unit and Kangaroo Mother Care admissions declined concurrently with these interventions (*57*). In South Africa, CHAMPS helped expand access to antenatal ultrasound services; ultrasound scans were performed for 696 pregnancies during June 2023–April 2024 (*58*). In addition, aspirin as a preventive treatment for pregnancy-related hypertensive disorders was added to the South African national formulary in response to CHAMPS data showing the burden of stillbirths and neonatal deaths related to these disorders. In Sierra Leone, CHAMPS findings that demonstrated that malnutrition was associated with 48% of deaths among children aged <5 years supported the country’s first domestic procurement of ready-to-use therapeutic food. The Sierra Leone government purchased 1,700 cartons (for US $99,000), and UNICEF secured an additional 1,700 cartons through a matching funding mechanism; supplies were distributed across all 16 districts (*59*). Moreover, CHAMPS findings on HIV infection supported an additional US $8 million investment from the U.S. President’s Emergency Plan for AIDS Relief (PEPFAR) for a population-based HIV impact assessment and the reinitiation of national HIV viral-load testing in Sierra Leone. These examples and others document changes in service delivery and resource allocation. Integrating CHAMPS findings with national surveillance, mortality review, and routine health information systems can help guide program response and strengthen accountability for achieving the United Nations’ goal of ending preventable deaths among newborns and children aged <5 years by 2030.

Implementation of CHAMPS also strengthened public health capacity. Site teams conducted bereavement-sensitive postmortem investigations in partnership with communities, demonstrating that MITS-based mortality surveillance can be feasible and acceptable when supported by sustained community engagement, transparent consent processes, and family-centered disclosure of results (*5*,*12*,*24*). Network investments strengthened laboratory, pathology, data, and analytic systems, including microbiology and molecular testing, histopathology, quality assurance, and standardized cause-of-death review (*15*,*16*,*43*,*60*). These capacities extend beyond the analyses in this report by supporting surveillance for priority pathogens, outbreak investigations, and local quality-improvement activities.

## Limitations

The findings in this report are subject to at least four limitations. First, CHAMPS catchment areas might not be nationally representative, and the analytic population was shaped by site selection, death notification, family consent, and MITS eligibility requirements. Because MITS required timely notification and specimen collection, deaths occurring in health care facilities were more likely to be investigated. Consequently, community deaths, deaths with delayed notification, deaths evaluated primarily through verbal autopsy, and certain injury-related deaths might be underrepresented (*47*,*61*).

Second, data completeness varied across sites and over time. Maternal and child clinical records, antenatal screening results, placental data, premortem diagnostics, and documentation of treatments were sometimes incomplete. These missing data could have limited assessment of upstream contributors to stillbirth, prematurity, fetal growth restriction, intrauterine hypoxia, congenital conditions, and chronic or noninfectious diseases. Certain maternal infections, chronic maternal conditions, child comorbidities, and structural abnormalities might therefore have been underdiagnosed or misclassified.

Third, pathogen attribution in postmortem surveillance is complex. CHAMPS used standardized laboratory, pathology, and DeCoDe procedures to distinguish pathogens considered causal from organisms detected incidentally or through contamination; however, attribution could still vary because of true epidemiologic differences, differences in case mix and enrollment, previous antimicrobial exposure, timing of specimen collection, and residual differences in interpretation across panels despite quality-assurance procedures (*13*,*14*). This broad surveillance summary presents pathogens included in the causal chain of death but does not provide detailed, case-level analyses of antimicrobial susceptibility, antibiotic access and use, treatment failure, or infection source. More detailed pathogen- and syndrome-specific CHAMPS analyses have addressed these problems for priority organisms and clinical syndromes, including *K. pneumoniae*, pneumonia, and neonatal bacterial infections (*17*,*22*,*28*,*36*). Therefore, aggregate pathogen findings in this report should be interpreted as an overall surveillance summary and considered in addition to organism-specific analyses of antimicrobial resistance, antibiotic treatment options, and community-associated versus health care–associated acquisition.

Finally, preventability assessments depended on available documentation and expert judgment. DeCoDe panels evaluated whether changes in health care–seeking behaviors, health care access, quality of care, or health care system performance within the local context might plausibly have averted the death, but these assessments were not designed to quantify the population-level impact of specific interventions. Preventability findings should therefore be interpreted as indicators of actionable gaps and opportunities for improvement, not as causal estimates of how many deaths would be prevented by any single intervention.

## Future Directions

CHAMPS demonstrates that standardized postmortem surveillance can generate actionable information for families, clinicians, health care systems, and public health programs. In the future, CHAMPS will measure changes in causes of death as health policies evolve and will adapt surveillance methods to align with changing country priorities, local governance, and mortality review and civil registration systems. Although the full CHAMPS model requires specialized infrastructure and trained personnel, select components (e.g., standardized MITS training, targeted sampling, tiered diagnostics, streamlined DeCoDe review, and structured linkage to perinatal and child death review) could be adapted for sentinel mortality surveillance in more locations, special studies, and outbreak investigations.

## Conclusion

These findings indicate that many fetal, neonatal, and child deaths in high-mortality settings remain preventable with timely, evidence-based interventions. Important contributors varied by age group and included perinatal asphyxia or hypoxia, sepsis, preterm birth complications, lower respiratory infections, congenital birth defects, malnutrition, malaria, and maternal conditions associated with stillbirth and neonatal death. Many of these contributors can be addressed through established maternal, newborn, and child health programs when delivered with sufficient quality, timeliness, and equity. By providing standardized, laboratory-supported mortality surveillance, CHAMPS has improved understanding of when, where, and why these deaths occur and has identified modifiable gaps in care and prevention. As countries work toward the United Nations’ targets for maternal, newborn, and child survival, integrating pathology, microbiology, epidemiology, and mortality review within national systems can help bridge diagnostic gaps, strengthen local capacity, and support prevention of avoidable deaths.

## Footnotes

* 45 C.F.R. part 46.102(l)(2), 21 C.F.R. part 56; 42 U.S.C. Sect. 241(d); 5 U.S.C. Sect. 552a; 44 U.S.C. Sect. 3501 et seq.

## References

[1] United Nations Inter-agency Group for Child Mortality Estimation. Levels & trends in child mortality: report 2023. New York, New York: UNICEF; 2024. https://data.unicef.org/wp-content/uploads/2024/03/UNICEF-2023-Child-Mortality-Report.pdf

[2] Bhutta ZA, Darmstadt GL, Haws RA, Yakoob MY, Lawn JE. Delivering interventions to reduce the global burden of stillbirths: improving service supply and community demand. BMC Pregnancy Childbirth 2009;9(Suppl 1):S7. 10.1186/1471-2393-9-S1-S7 19426470 PMC2679413

[3] Madewell ZJ, Whitney CG, Velaphi S, ; Child Health and Mortality Prevention Surveillance network. Prioritizing health care strategies to reduce childhood mortality. JAMA Netw Open 2022;5:e2237689. 10.1001/jamanetworkopen.2022.37689 36269354 PMC9587481

[4] Rampatige R, Mikkelsen L, Hernandez B, Riley I, Lopez AD. Systematic review of statistics on causes of deaths in hospitals: strengthening the evidence for policy-makers. Bull World Health Organ 2014;92:807–16. 10.2471/BLT.14.137935 25378742 PMC4221770

[5] Salzberg NT, Sivalogan K, Bassat Q, ; Child Health and Mortality Prevention Surveillance (CHAMPS) Methods Consortium. Mortality surveillance methods to identify and characterize deaths in Child Health and Mortality Prevention Surveillance network sites. Clin Infect Dis 2019;69(Suppl 4):S262–73. 10.1093/cid/ciz599 31598664 PMC6785672

[6] Leulseged H, Bethencourt C, Igunza KA, ; Child Health and Mortality Prevention Surveillance (CHAMPS) network. Clinicopathological discrepancies in the diagnoses of childhood causes of death in the CHAMPS network: an analysis of antemortem diagnostic inaccuracies. BMJ Paediatr Open 2024;8:e002654. 10.1136/bmjpo-2024-002654 39032935 PMC11409330

[7] Taylor AW, Blau DM, Bassat Q, ; CHAMPS Consortium. Initial findings from a novel population-based child mortality surveillance approach: a descriptive study. Lancet Glob Health 2020;8:e909–19. 10.1016/S2214-109X(20)30205-9 32562647 PMC7303945

[8] Bassat Q, Blau DM, Ogbuanu IU, ; Child Health and Mortality Prevention Surveillance (CHAMPS) network. Causes of death among infants and children in the Child Health and Mortality Prevention Surveillance (CHAMPS) network. JAMA Netw Open 2023;6:e2322494. 10.1001/jamanetworkopen.2023.22494 37494044 PMC10372710

[9] Mahtab S, Madhi SA, Baillie VL, ; CHAMPS Consortium. Causes of death identified in neonates enrolled through Child Health and Mortality Prevention Surveillance (CHAMPS), December 2016–December 2021. PLOS Glob Public Health 2023;3:e0001612. 10.1371/journal.pgph.0001612 36963040 PMC10027211

[10] Otieno P, Akelo V, Khagayi S, Acceptability of minimally invasive autopsy by community members and healthcare workers in Siaya and Kisumu counties, western Kenya, 2017–2018. PLOS Glob Public Health 2023;3:e0001319. 10.1371/journal.pgph.0001319 37747874 PMC10519588

[11] Rakislova N, Fernandes F, Lovane L, Standardization of minimally invasive tissue sampling specimen collection and pathology training for the Child Health and Mortality Prevention Surveillance network. Clin Infect Dis 2019;69(Suppl 4):S302–10. 10.1093/cid/ciz565 31598667 PMC6785668

[12] O’Mara Sage E, Munguambe KR, Blevins J, Investigating the feasibility of child mortality surveillance with postmortem tissue sampling: generating constructs and variables to strengthen validity and reliability in qualitative research. Clin Infect Dis 2019;69(Suppl 4):S291–301. 10.1093/cid/ciz564 31598657 PMC6785679

[13] Breiman RF, Blau DM, Mutevedzi P, ; CHAMPS Consortium. Postmortem investigations and identification of multiple causes of child deaths: an analysis of findings from the Child Health and Mortality Prevention Surveillance (CHAMPS) network. PLoS Med 2021;18:e1003814. 10.1371/journal.pmed.1003814 34591862 PMC8516282

[14] Blau DM, Caneer JP, Philipsborn RP, Overview and development of the Child Health and Mortality Prevention Surveillance Determination of Cause of Death (DeCoDe) process and DeCoDe diagnosis standards. Clin Infect Dis 2019;69(Suppl 4):S333–41. 10.1093/cid/ciz572 31598661 PMC6785670

[15] Diaz MH, Waller JL, Theodore MJ, Development and implementation of Multiplex TaqMan array cards for specimen testing at Child Health and Mortality Prevention Surveillance site laboratories. Clin Infect Dis 2019;69(Suppl 4):S311–21. 10.1093/cid/ciz571 31598666 PMC7108207

[16] Martines RB, Ritter JM, Gary J, Pathology and telepathology methods in the Child Health and Mortality Prevention Surveillance network. Clin Infect Dis 2019;69(Suppl 4):S322–32. 10.1093/cid/ciz579 31598668

[17] Verani JR, Blau DM, Gurley ES, Child deaths caused by *Klebsiella pneumoniae* in sub-Saharan Africa and South Asia: a secondary analysis of Child Health and Mortality Prevention Surveillance (CHAMPS) data. Lancet Microbe 2024;5:e131–41. 10.1016/S2666-5247(23)00290-2 38218193 PMC10849973

[18] Blau DM, Baillie VL, Els T, ; CHAMPS Consortium. Deaths attributed to respiratory syncytial virus in young children in high-mortality rate settings: report from Child Health and Mortality Prevention Surveillance (CHAMPS). Clin Infect Dis 2021;73(Suppl_3):S218–28. 10.1093/cid/ciab509 34472577 PMC8411256

[19] Mahtab S, Madewell ZJ, Baillie V, ; CHAMPS Consortium. Etiologies and comorbidities of meningitis deaths in children under 5 years in high-mortality settings: insights from the CHAMPS network in the post-pneumococcal vaccine era. J Infect 2024;89:106341. 10.1016/j.jinf.2024.106341 39521254 PMC11624489

[20] Ajanovic S, Madewell ZJ, El Arifeen S, ; Child Health and Mortality Prevention Surveillance (CHAMPS) Consortium. Neurological symptoms and cause of death among young children in low- and middle-income countries. JAMA Netw Open 2024;7:e2431512. 10.1001/jamanetworkopen.2024.31512 39226053 PMC11372484

[21] Mahtab S, Madewell ZJ, Madhi SA, ; CHAMPS Consortium. Stillbirths and neonatal deaths caused by group B *Streptococcus* in Africa and South Asia identified through Child Health and Mortality Prevention Surveillance (CHAMPS). Open Forum Infect Dis 2023;10:ofad356. 10.1093/ofid/ofad356 37674633 PMC10478157

[22] Ojulong J, Gebru GN, Duduyemi B, Prevalence of antimicrobial resistance in *Klebsiella pneumoniae, Enterobacter cloacae*, and *Escherichia coli* isolates among stillbirths and deceased under-five children in Sierra Leone: data from the Child Health and Mortality Prevention Surveillance sites from 2019 to 2022. Microorganisms 2024;12:1657. 10.3390/microorganisms12081657 39203499 PMC11356759

[23] Cunningham SA, Shaikh NI, Nhacolo A, Health and demographic surveillance systems within the Child Health and Mortality Prevention Surveillance network. Clin Infect Dis 2019;69(Suppl 4):S274–9. 10.1093/cid/ciz609 31598663 PMC6785673

[24] Blevins J, O’Mara Sage E, Kone A, Using participatory workshops to assess alignment or tension in the community for minimally invasive tissue sampling prior to start of child mortality surveillance: lessons from 5 sites across the CHAMPS network. Clin Infect Dis 2019;69(Suppl 4):S280–90. 10.1093/cid/ciz563 31598665 PMC6785692

[25] Rahman A, Lee KH, El Arifeen S, Causes of stillbirth in sub-Saharan Africa and South Asia: findings from Child Health and Mortality Prevention Surveillance, 2016–2023. medRxiv [Preprint posted online September 12, 2025]. https://www.medrxiv.org/content/10.1101/2025.09.09.25335469v1 10.1101/2025.09.09.25335469

[26] Madewell ZJ, Keita AM, Das PM, ; Child Health and Mortality Prevention Surveillance network. Contribution of malnutrition to infant and child deaths in sub-Saharan Africa and South Asia. BMJ Glob Health 2024;9:e017262. 10.1136/bmjgh-2024-017262 39638608 PMC11624724

[27] Das PM, Madewell ZJ, Blau DM, ; Child Health and Mortality Prevention Surveillance network. Importance of postmortem anthropometric evaluation in defining the role of malnutrition as a cause of infant and child deaths in sub-Saharan Africa and South Asia: a cohort study. BMJ Open 2025;15:e089874. 10.1136/bmjopen-2024-089874 39961713 PMC11836817

[28] Mahtab S, Blau DM, Madewell ZJ, ; CHAMPS Consortium. Post-mortem investigation of deaths due to pneumonia in children aged 1–59 months in sub-Saharan Africa and South Asia from 2016 to 2022: an observational study. Lancet Child Adolesc Health 2024;8:201–13. 10.1016/S2352-4642(23)00328-0 38281495 PMC10864189

[29] Velaphi S, Madewell ZJ, Tippett-Barr B, Investigating the role of cytomegalovirus as a cause of stillbirths and child deaths in low and middle-income countries through postmortem minimally invasive tissue sampling. Clin Infect Dis 2026;82:326–36. 10.1093/cid/ciaf098 40059623 PMC13017237

[30] Mutevedzi PC, Madewell ZJ, Kotloff KL, ; CHAMPS Consortium. Use of minimally invasive tissue sampling to determine the contribution of diarrheal diseases to under-five mortality and associated co-morbidities and co-infections in children with fatal diarrheal diseases in Africa and Bangladesh. PLOS Glob Public Health 2025;5:e0004772. 10.1371/journal.pgph.0004772 40561131 PMC12193650

[31] Ogbuanu IU, Otieno K, Varo R, ; CHAMPS consortium. Burden of child mortality from malaria in high endemic areas: results from the CHAMPS network using minimally invasive tissue sampling. J Infect 2024;88:106107. 10.1016/j.jinf.2024.01.006 38290664 PMC13019364

[32] Mandomando I, Madewell ZJ, Mutevedzi PC, ; Child Health and Mortality Prevention Surveillance network. Post-mortem characterisation of HIV-associated under-5 deaths in the CHAMPS network: population-based mortality surveillance. Lancet HIV 2026;13:e247–57. 10.1016/S2352-3018(25)00330-3 41759541

[33] Assefa N, Scott A, Madrid L, Comparison of causes of stillbirth and child deaths as determined by verbal autopsy and minimally invasive tissue sampling. PLOS Glob Public Health 2024;4:e0003065. 10.1371/journal.pgph.0003065 39074089 PMC11285936

[34] World Health Organization. WHO recommendations on interventions to improve preterm birth outcomes. Geneva, Switzerland: World Health Organization; 2015. https://www.who.int/publications/i/item/9789241508988 26447264

[35] de Bernis L, Kinney MV, Stones W, ; Lancet Ending Preventable Stillbirths Series Study Group; Lancet Ending Preventable Stillbirths Series Advisory Group. Stillbirths: ending preventable deaths by 2030. Lancet 2016;387:703–16. 10.1016/S0140-6736(15)00954-X 26794079

[36] Alam M, Lee KH, Baillie V, Post-mortem characterisation of pathogen-specific causes of infection-related deaths in African and South Asian neonates: a prospective, observational, multicentre study. Lancet Infect Dis 2026. Epub May 13, 2026. https://www.thelancet.com/journals/laninf/article/PIIS1473-3099(26)00136-2/fulltext 10.1016/S1473-3099(26)00136-2 42127967

[37] Bhutta ZA, Das JK, Bahl R, ; Lancet Newborn Interventions Review Group; Lancet Every Newborn Study Group. Can available interventions end preventable deaths in mothers, newborn babies, and stillbirths, and at what cost? Lancet 2014;384:347–70. 10.1016/S0140-6736(14)60792-3 24853604

[38] Gupta PM, Madewell ZJ, Gannon BM, Hepatic vitamin A concentrations and association with infectious causes of child death. J Pediatr 2024;265:113816. 10.1016/j.jpeds.2023.113816 37931699 PMC10869935

[39] Varo R, Cole K, Madewell ZJ, ; Child Health and Mortality Prevention Surveillance (CHAMPS) network. Deaths with preceding hospitalisations within 180 days in eight countries in sub-Saharan Africa and South Asia: a secondary descriptive analysis of the Child Health and Mortality Prevention Surveillance (CHAMPS) network. BMJ Open 2026;16:e106095. 10.1136/bmjopen-2025-106095 41877327 PMC13034342

[40] World Health Organization. Integrated management of childhood illness. Geneva, Switzerland: World Health Organization; 2026. https://www.who.int/teams/maternal-newborn-child-adolescent-health-and-ageing/child-health/integrated-management-of-childhood-illness

[41] Lawn JE, Lee AC, Kinney M, Two million intrapartum-related stillbirths and neonatal deaths: where, why, and what can be done? Int J Gynaecol Obstet 2009;107(Suppl 1):S5–19. 10.1016/j.ijgo.2009.07.016 19815202

[42] Ellis M, Azad K, Banerjee B, Intrapartum-related stillbirths and neonatal deaths in rural Bangladesh: a prospective, community-based cohort study. Pediatrics 2011;127:e1182–90. 10.1542/peds.2010-0842 21502233

[43] Kamara S, Kowuor D, Samura SS, Laying the foundations for high-quality mortality surveillance in Sierra Leone: early learnings from the Child Health and Mortality Prevention Surveillance (CHAMPS) network. Gates Open Res 2024;8:98. 10.12688/gatesopenres.15986.1

[44] World Health Organization; UNICEF. Every Newborn: an action plan to end preventable deaths. Geneva, Switzerland: World Health Organization; 2014. https://www.who.int/publications/i/item/9789241507448

[45] World Health Organization. Maternal, newborn, child and adolescent health and ageing data portal. Geneva, Switzerland: World Health Organization; 2021. https://platform.who.int/data/maternal-newborn-child-adolescent-ageing

[46] Vyas KJ, Muir JA, Madewell ZJ, ; CHAMPS Network. Major causes of perinatal and paediatric mortality in sub-Saharan Africa and South Asia: adjustment for selection bias in the CHAMPS network. Paediatr Perinat Epidemiol 2025;39:698–710. 10.1111/ppe.70067 40905263 PMC12658314

[47] Blencowe H, Cousens S, Kamb M, Berman S, Lawn JE. Lives Saved Tool supplement detection and treatment of syphilis in pregnancy to reduce syphilis related stillbirths and neonatal mortality. BMC Public Health 2011;11(Suppl 3):S9. 10.1186/1471-2458-11-S3-S9 21501460 PMC3231915

[48] Mwansa-Kambafwile J, Cousens S, Hansen T, Lawn JE. Antenatal steroids in preterm labour for the prevention of neonatal deaths due to complications of preterm birth. Int J Epidemiol 2010;39(Suppl 1):i122–33. 10.1093/ije/dyq029 20348115 PMC2845868

[49] Rees CA, Igunza KA, Madewell ZJ, ; Child Health and Mortality Prevention Surveillance network. Provider adherence to clinical care recommendations for infants and children who died in seven low- and middle-income countries in the Child Health and Mortality Prevention Surveillance (CHAMPS) network. EClinicalMedicine 2023;63:102198. 10.1016/j.eclinm.2023.102198 37692079 PMC10484959

[50] Garcia Gomez E, Igunza KA, Madewell ZJ, ; Child Health and Mortality Prevention Surveillance network. Identifying delays in healthcare seeking and provision: the Three Delays-in-Healthcare and mortality among infants and children aged 1–59 months. PLOS Glob Public Health 2024;4:e0002494. 10.1371/journal.pgph.0002494 38329969 PMC10852234

[51] Rahman A, Ray M, Madewell ZJ, ; Child Health and Mortality Prevention Surveillance (CHAMPS) network. Adherence to perinatal asphyxia or sepsis management guidelines in low- and middle-income countries. JAMA Netw Open 2025;8:e2510790. 10.1001/jamanetworkopen.2025.10790 40377940 PMC12084843

[52] World Health Organization. WHO position paper: pneumococcal conjugate vaccines in infants and children aged <5 years—September 2025. Geneva, Switzerland: World Health Organization; 2025. https://www.who.int/publications/i/item/who-wer10039-411-437

[53] World Health Organization. Country guidance for planning triple elimination of mother-to-child transmission of HIV, syphilis and hepatitis B virus programmes. Geneva, Switzerland: World Health Organization; 2025. https://www.who.int/publications/i/item/9789240112490

[54] Madrid L, Vyas KJ, Kancherla V, ; CHAMPS Consortium. Neural tube defects as a cause of death among stillbirths, infants, and children younger than 5 years in sub-Saharan Africa and Southeast Asia: an analysis of the CHAMPS network. Lancet Glob Health 2023;11:e1041–52. 10.1016/S2214-109X(23)00191-2 37271162 PMC10282076

[55] Igunza KA, Tippett Barr BA, Madewell ZJ, Understanding the role of parental decision-making and quality of clinical care in under-5 mortality in Western Kenya: an application of the Three Delays model. BMC Health Serv Res 2026. Epub May 21, 2026. https://link.springer.com/article/10.1186/s12913-026-14634-8 10.1186/s12913-026-14634-8 PMC13474843 42169072

[56] Ngere SH, Akelo V, Ondeng’e K, Traditional medicine beliefs and practices among caregivers of children under five years—the Child Health and Mortality Prevention Surveillance (CHAMPS), western Kenya: a qualitative study. PLoS One 2022;17:e0276735. 10.1371/journal.pone.0276735 36322582 PMC9629611

[57] Sigu B, Akoth L, Ojwang E, CHAMPS collaborates with local MoH to reduce neonatal mortality by promoting Kangaroo Mother Care (KMC) and Enhancing Emergency Obstetric Care (EmOC) in Siaya, western Kenya. Atlanta, GA: Emory University, Child Health and Mortality Prevention Surveillance; 2024. https://champshealth.org/case_study/champs-collaborates-with-local-moh-to-reduce-neonatal-mortality-by-promoting-kangaroo-mother-care-kmc-and-enhancing-emergency-obstetric-care-emoc-in-siaya-western-kenya/

[58] Ntimani B, Morifi M, Dangor Z, Madhi S, Mutevedzi PC. Improving pregnancy health and outcomes through provision of ultrasound during pregnancy: collaborative efforts between the South Africa MoH and WITS-VIDA. Atlanta, GA: Emory University, Child Health and Mortality Prevention Surveillance; 2024. https://champshealth.org/case_study/improving-pregnancy-health-and-outcomes-through-provision-of-ultrasound-during-pregnancy-collaborative-efforts-between-the-south-africa-moh-and-wits-vida/

[59] Ogbuanu IU, Kaluma EM, Ogbuanu C, Mutevedzi PC. Data dissemination levers: engaging partners and stakeholders to translate CHAMPS data into actionable public health policies and interventions—two success stories at the CHAMPS SL site. Atlanta, GA: Emory University, Child Health and Mortality Prevention Surveillance; 2024. https://champshealth.org/case_study/data-dissemination-levers-engaging-partners-and-stakeholders-to-translate-champs-data-into-actionable-public-health-policies-and-interventions-two-success-stories-at-the-champs-sl-site

[60] Sacoor C, Vitorino P, Nhacolo A, Child Health and Mortality Prevention Surveillance (CHAMPS): Manhiça site description, Mozambique. Gates Open Res 2024;7:4. 10.12688/gatesopenres.13931.3 39233704 PMC11374382

[61] Rahman A, Alonge O, Bhuiyan AA, Epidemiology of drowning in Bangladesh: an update. Int J Environ Res Public Health 2017;14:488. 10.3390/ijerph14050488 28475138 PMC5451939

